# Restoration of Miro1’s N-terminal GTPase function alleviates prenatal stress-induced mitochondrial fission via Drp1 modulation

**DOI:** 10.1186/s12964-025-02172-5

**Published:** 2025-04-02

**Authors:** Gee Euhn Choi, Ji Yong Park, Mo Ran Park, Chang Woo Chae, Young Hyun Jung, Jae Ryong Lim, Jee Hyeon Yoon, Ji Hyeon Cho, Ho Jae Han

**Affiliations:** 1 Department of Veterinary Physiology, College of Veterinary Medicine, Research Institute for Veterinary Science, BK21 FOUR Future Veterinary Medicine Leading Education & Research Center, Seoul National University, Seoul, 08826 South Korea; 2 Laboratory of Veterinary Biochemistry, College of Veterinary Medicine and Veterinary Medical Research Institute, Jeju National University, Jeju, 63243 South Korea; 3 Interdisciplinary Graduate Program in Advanced Convergence Technology & Science, Jeju National University, Jeju, 63243 South Korea; 4 Department of Physiology and Medical Science, College of Medicine and Brain Research Institute, Chungnam National University, Daejeon, 35015 South Korea; 5 Department of Physiology, College of Medicine, Soonchunhyang University, Cheonan, 31151 Republic of Korea

**Keywords:** ER-mitochondria contacts, Miro, Mitochondrial dynamics, Neurodegeneration, Prenatal glucocorticoid

## Abstract

**Background:**

Prenatal stress exposure irreversibly impairs mitochondrial dynamics, including mitochondrial trafficking and morphology in offspring, leading to neurodevelopmental and neuropsychiatric disorders in adulthood. Thus, understanding the molecular mechanism controlling mitochondrial dynamics in differentiating neurons is crucial to prevent the prenatal stress-induced impairments in behavior. We investigated the interplay between mitochondrial transport and fusion/fission in differentiating neurons exposed to prenatal stress, leading to ensuing behavior impairments, and then tried to identify the primary regulator that modulates both phenomena.

**Methods:**

We used primary hippocampal neurons of mice exposed to prenatal stress and human induced-pluripotent stem cell (hiPSC)-derived neurons, for investigating the impact of glucocorticoid on mitochondrial dynamics during differentiation. For constructing mouse models, we used AAV vectors into mouse pups exposed to prenatal stress to regulate protein expressions in hippocampal regions.

**Results:**

We first observed that prenatal exposure to glucocorticoids induced motility arrest and fragmentation of mitochondria in differentiating neurons derived from mouse fetuses (E18) and human induced pluripotent stem cells (hiPSCs). Further, glucocorticoid exposure during neurogenesis selectively downregulated Miro1 and increased Drp1 phosphorylation (Ser616). MIRO1 overexpression restored mitochondrial motility and increased intramitochondrial calcium influx through ER-mitochondria contact (ERMC) formation. Furthermore, we determined that the N-terminal GTPase domain of Miro1 plays a critical role in ERMC formation, which then decreased Drp1 phosphorylation (Ser616). Similarly, prenatal corticosterone exposure led to impaired neuropsychiatric and cognitive function in the offspring by affecting mitochondrial distribution and synaptogenesis, rescued by Miro1^WT^, but not N-terminal GTPase active form Miro1^P26V^, expression.

**Conclusion:**

Prenatal glucocorticoid-mediated Miro1 downregulation contributes to dysfunction in mitochondrial dynamics through Drp1 phosphorylation (Ser616) in differentiating neurons.

**Supplementary Information:**

The online version contains supplementary material available at 10.1186/s12964-025-02172-5.

## Background

Excess cortisol from maternal stressors can permeate the placental barrier, permanently reprogramming the hypothalamus–pituitary–adrenal (HPA) axis during pregnancy, predisposing offspring to neurodevelopmental disorders later in life [1, 2]. Such exposure leads to irreversible alterations, contrasting with the more transient, reversible effects typically seen in adult stress responses. This irreversibility manifests in long-term changes in protein expression patterns and cellular behavior that fundamentally differ from adult models, underscoring the critical impact of prenatal stress. Stress-induced elevations in glucocorticoids during neurogenesis disrupt mitochondrial dynamics, affecting key aspects like morphology and transport, which typically precede hallmark neurodegenerative deficits, including impaired neuronal migration and synaptic refinement [3–5]. The developing brain, rich in neural stem cells (NSCs), is particularly sensitive to mitochondrial disruptions due to its unique metabolic demands. Mitochondrial morphology and distribution thereby undergo rapid transitions during this stage, with increased fragmentation which is rather typically seen in neurodegenerative phenotypes in terminally differentiated neurons [6]. The precise regulation of mitochondrial dynamics during this critical period is essential for neuronal plasticity mediated by tremendous energy from mitochondria, as developing neurons are more vulnerable to stress impacts compared to mature neurons [6–8]. Any disruptions in neurogenesis during this stage can lead to permanent physiological defects, as these differentiated neurons are retained for life, carrying forward any stress-induced impairments.

Mitochondrial transport and fusion/fission have been studied as separate processes. However, recent findings indicate a significant interplay, as fission/fusion machinery interacts with mitochondrial transport proteins, suggesting a coordinated regulation that is particularly relevant during neurodevelopment under stress [9]. Dysregulation in this coordination, especially under prenatal stress conditions, can have long-lasting effects [10, 11]. For example, mutations in key fusion-related genes such as *mfn2* disrupt mitochondrial distribution and transport in neurons, as seen in cultured rodent and fly models [10, 12, 13]. Impairments in mitochondrial Rho GTPase 1 (Miro1), which participates in mitochondrial trafficking, lead to disrupted mitophagy and age-related hyperfusion, impacting neuroblast formation and neuronal integrity [5, 14]. These studies suggest that a primary key molecule can disrupt various aspects of mitochondrial dynamics, causing significant damage to neuronal function. Therefore, this study aims to elucidate the key primary molecule that coordinates the crosstalk between mitochondrial transport and fusion/fission in differentiating neurons affected by prenatal stress. By targeting and modulating this protein, we aim to develop therapeutic strategies that could mitigate the long-term neurological risks imposed on offspring by prenatal stress.

To address this, we used mouse primary hippocampal neurons to explore how prenatal exposure to stress-induced glucocorticoid disrupt mitochondrial transport and fusion/fission dynamics. Additionally, human-induced pluripotent stem cell (hiPSC)-derived NSCs exposed to elevated cortisol during differentiation allowed us to model human-like stress conditions. Finally, ICR mouse pups exposed to stress-induced corticosterone at embryonic day 14 (E14) to stimulate prenatal stress and assess subsequent damaging effects of mitochondrial dysfunction on cognitive and neuropsychiatric behavior. Through these approaches, this study provides a comprehensive investigation of prenatal stress-induced mitochondrial dysfunction and its role in neurodevelopmental disorders, using both in vitro and in vivo models.

## Materials and methods

### Cell cultures

The iPSCs were obtained from the National Stem Cell Bank of Korea and Korean Cell Line Bank (KSCBi005-A). iPSCs were cultured at 100,000 cells on plates coated with recombinant human vitronectin (#A14700, Thermo Fisher) and treated with neural induction media (#A1647801, Thermo Fisher) to induce neuronal stem cells (NSCs). The cells were replated on dishes coated with Geltrex (#A1413201, Thermo Fisher) and grown in neural differentiation media Neurobasal medium (#21103, Thermo Fisher) supplemented with 2% B27 (#17504, Thermo Fisher) and 1% GlutaMax-1 (#35050, Thermo Fisher) and maintained in a 37 ℃, 5% CO_2_ incubator with humidified atmosphere for 10 days. For establishing the neurons exposed to prenatal stress, we treated cortisol during neurogenesis [15].

Mouse hippocampal neurons from E18 embryos from female mice exposed to maternal stressor corticosterone (10 mg/kg) for growing neurons with prenatal stress were cultured according to a modified protocol [6, 16], and performed in compliance with the approval of the Institutional Animal Care and Use Committee of Seoul National University (SNU-230522-3). Briefly, hippocampal neurons were cultured at low density of 10,000 cells on poly-D-lysine-coated coverslips (#GG-18-15-PDL, Neuvitro, Vancouver, WA, USA) with cortical rings of neurons and glia or at high density of 100,000 cells on six-well plates coated with poly-D-lysine in neurobasal medium supplemented with 2% B27 and 0.25% GlutaMax-1, using pups from female mice exposed to maternal stressor.

### Transfection of small interfering RNA (siRNA) for gene Silencing

Neurons were grown until approximately 60% confluency on the plate. Prior to corticosterone and cortisol treatment, differentiating neurons were incubated with a mixture of 25 nM indicated siRNA and the turbofect transfection reagent (#R0531, Thermo Fisher) for 24 h, following to the manufacturer’s instructions. The siRNA specific for human *DNM1L* and mouse *dnm1l*, and siRNA specific for nontargeting (NT) were purchased from Bioneer Corporation (Daejeon, Korea).

### Lentivirus and Adeno-Associated virus production and infection

Neurons were grown until approximately 60% confluency on the plate. Prior to corticosterone and cortisol treatment, differentiating neurons were transfected with viral vector. For this study, lentivirus and AAV serotype 2 were used. Human Miro1 (wild-type, P13V, and E208K/E328K) coding sequences were cloned into a lentiviral vector under EF1A promoter. All wild-type or mutant types of pLV-EGFP: T2A: Puro-EF1A-RHOT1 vectors were produced by Vectorbuilder (Chicago, IL, USA). Transduction of lentiviral vectors were done in human iPSC-derived neurons with 20 multiplicity of infection (MOI). Mouse Miro1 (wild-type, P26V) coding sequences were cloned into an AAV2 under the CMV promoter. Wild-type or mutant type of pAAV-CMV-EGFP: WPRE vectors were produced by Vectorbuilder. Transduction of AAVs were done in mouse primary hippocampal neurons with 20,000 MOI. Two days after transduction, puromycin (2 µg/ml) was applied for selection of transduced cells.

### Transfection of plasmid DNA

According to the manufacturer’s instructions, we used mCherry-Drp1(the mCh-Drp1 was a gift from Gia Voeltz (Addgene plasmid # 49152; http://n2t.net/addgene:49152; RRID: Addgene_49152)). The mCherry was a gift from Rob Parton (Addgene plasmid # 176016; http://n2t.net/addgene:176016; RRID: Addgene_176016). Neurons were incubated with the mCherry control and mCherry-Drp1 plasmids were transfected using lipofectamine 3000 (Thermo Fisher) in accordance with the manufacturer’s protocol.

### Western blot analysis

The samples were lysed with the EzRIPA buffer (#WSE- 7420, ATTO, Tokyo, Japan) containing protease and phosphatase inhibitors. Cell debris was removed by centrifugation (13,000 × g at 4 °C, 30 min). Protein determination was done by a bicinchoninic acid quantification assay kit (Thermo Fisher). Equal amount of sample proteins (1–5 µg) were subjected to 8–12% SDS-PAGE and transferred to a polyvinylidene fluoride (PVDF) membrane. Then, the membranes were blocked with 5% bovine serum albumin (Sigma Chemical Company) or 5% skim milk (Gibco) in tris-buffered saline containing 0.2% Tween-20 (TBST; 150 mM NaCl, 10 mM Tris–HCl (pH 7.6), 0.1% Tween-20) solution for 30 min. After washing with TBST three times, the membranes were probed with the primary antibody at 4 °C overnight. The primary antibodies used in immunocytochemistry were listed as follow: Fis1 (#sc-376447), GPR75 (#sc-164538), Miro1 (#sc-398520), Mfn1 (#sc-166644), Mfn2 (#sc-100560), OPA1 (#sc-367890), VDAC1 (#sc-390996), and β-actin (#sc-47778), IP3R (#ab5804), Synaptophysin (#ab32127), and TOMM20 (#ab56783), Drp1 (#NB110-55288), Kinesin 1 (#NB500-580), and Miro2 (#NBP1-88982), MCU (#14997S), and p-DRP1 (Ser616) (#3455S), Miro1 (#TA349118), and α-tubulin (#T6074). Next, the membranes were washed three times with TBST, followed by incubation with the HRP-conjugated secondary antibodies capturing mouse, rabbit, and goat IgG (Thermo Fisher) at room temperature for 2 h. The protein complexes were detected with chemiluminescence solution (BioRad, Hercules, CA, USA) followed by exposure of blots to Chemidoc™ XRS + system with Lab™ software (Bio-Rad), and the densitometry analysis for quantification was performed using Image J software (developed by Wayne Rasband, National Institutes of Health, Bethesda, MD, USA).

### Co‑immunoprecipitation

Primary antibodies were immobilized with SureBeads™ protein G magnetic beads (#1614023, BioRad). Immobilized magnetic beads were incubated with the total lysates of cells (300 µg) at 4 °C overnight. Magnetic beads were pulled down by a magnet and then acquired. The antibody-bound protein was collected by incubation in elution buffer (Thermo Fisher). Protein analysis was performed by western blot where anti-mouse or -rabbit IgG antibody was used as a negative control.

### Synaptosome isolation

Synaptosomes from the mouse hippocampus and neurons derived from neural stem cells (NSCs) were extracted using Syn-Per synaptic protein extraction reagent (#87793, Thermo Fisher). The hippocampus and neurons were homogenized with a Dounce grinder, employing 20 slow strokes. Subsequently, the homogenates underwent centrifugation at 1200 × g for 10 min at 4 °C. After discarding the pellet, supernatant was centrifuged at 15,000 × g for 20 min at 4 °C. The pellet that remained after this process is designated as the ‘synaptosome’.

### Immunocytochemistry

Cultured cells were fixed with 4% paraformaldehyde for 15 min at room temperature and then incubated in 0.1% Triton X for 5 min. Cells were placed in 5% normal goat serum (NGS) in PBS for 1 h. Next, the cells were incubated overnight at 4 °C with primary antibody dissolved in 5% NGS. The primary antibodies used in immunocytochemistry were listed as follow: Synaptophysin (#ab32127, Abcam), PSD95 (#MAB1596, Sigma Chemical Company), PSD95 (#3409, Cell Signaling Technology), Miro1 (#TA349118, OriGene Technologies), Kinesin (#NB500-580, Novus Biologicals), α-tubulin (#T6074, Sigma Chemical Company), IP3R (#ab5804, Abcam), TOMM20 (#ab56783, Abcam), Calnexin (#sc-23954, Santa Cruz Biotechnology), VDAC1 (#sc-390996, Santa Cruz Biotechnology), LAMP1 (#9091S, Cell Signaling Technology), DCX (#PA-17428, Thermo Fisher), β-III-tubulin (#MAB1195, R&D System). After being washed with PBS, the cells were incubated for 2 h at room temperature with Alexa Fluor™ secondary antibody (Thermo Fisher) as appropriate. Images were acquired by confocal microscope system (Carl Zeiss, LSM710) and super-resolution radial fluctuations (SRRF) imaging system (Andor Technology, Belfast, UK). The fluorescent intensity analysis and co-localization analysis with Pearson’s correlation coefficient were performed with Fiji software (developed by Wayne Rasband, National Institutes of Health, Bethesda, MD, USA).

### Analysis of mitochondrial morphology

After staining with MitoTracker Red (MTR) or MitoTracker Green (MTG), cells were visualized with microscopy. The analysis was performed using Fiji software. Mitochondrial morphology was analyzed as described following a modified protocol [17]. Briefly, individual mitochondria particles were analyzed for circularity (Form factor = (perimeter)^2^/(4 π × area)) and aspect ratio (lengths ratio of major and minor axis) after thresholding.

### Measurements of intracellular calcium

The pH-sensitive fluorescent probe Fluo-4 AM (#F14201, Thermo Fisher) exhibiting fluorescence upon binding Ca^2+^ were used for measuring intracellular calcium. Measurements were performed according to the manufacturer’s instructions. After incubation with 3 µM of Fluo-4 at 37 ℃ for 1 h, cells were stabilized and exposed to agents. Fluo-4-stained cells were measured by a flow cytometer (CytoFlex; Beckman Coulter, CA, USA).

### Measurements of MtROS, mitochondrial membrane potential, and mitochondrial calcium

MitoSOX™ (#M36008, Thermo Fisher), tetramethylrhodamine ethyl ester (TMRE) (#87917, Sigma Chemical Company), and Rhod-2 AM (#R1244, Invitrogen) were used to determine mtROS, mitochondrial membrane potential, and mitochondria calcium respectively. Cells were washed once with PBS and incubated with 2.5 µM MitoSOX™ for 10 min at 37 ℃, 500 nM TMRE for 30 min, and 3 µM Rhod-2 for 1 h min at 37 ℃. After being washed with PBS three times, cells were treated with 0.05% trypsin for 3 min and centrifuged at 1,500 × g for 5 min. Harvested cells were suspended in 400 µL PBS. Fluorescence intensities of MitoSOX™, and TMRE were detected by a flow cytometer. To measure mitochondrial calcium dynamics through imaging, cells were incubated with 3 µM Rhod-2 for 1 h and then washed with calcium-free media. Images were acquired for 5 min at 2 s intervals using Eclipse Ts2™ fluorescence microscopy (Nikon, Tokyo, Japan) while ionomycin (10 µM) was added at 10 s after start. The change of mitochondrial Ca^2+^ dynamics was quantified as the ratio of fluorescence value (f) in a region of interest after ionomycin treatment to the fluorescence prior to ionomycin treatment (f_0_).

### Mitochondrial stress test assay

Oxygen consumption rate (OCR) under mitochondrial stress test assay was performed using the XF Cell Mito Stress Test Kit (#103015-100), and the data were required from Seahorse XF24 Extracellular Flux Analyzer (Agilent Technologies, Santa Clara, CA, USA), following the manufacturer’s instructions. NSC-derived neurons were cultured in XF24 cell culture microplate. Oligomycin, FCCP, and antimycin A/rotenone mixture were treated to cell culture to determine the mitochondrial respiration profiles including basal/maximal respiration, and ATP production.

### Live imaging of mitochondrial trafficking

Live imaging of mitochondrial transport in hippocampal neuron or human iPSC-derived neuron were performed by using an upright Zeiss LSM700 (Carl Zeiss, Oberkochen, Germany) confocal microscope (with a 63X immersion objective) with modification as described [18]. Cells were placed on a platform with controlled temperature (37 ℃) and air ventilation. Axonal images were acquired every 5 s for at least 5 min without averaging. Kymographs were generated from time-lapse movies by using the Velocity Measurement Tool (VMT), which is a freely available Image J macro. To reduce motion noise, the videos were stabilized offline using the Bio-Formats plugin in Fiji/Image J. Kymographs were generated individual axonal segments longer than 50 mm were selected, and a line following the path of the mitochondria was traced on an average-intensity stack image using the segmented line tool.

### Fluorescence imaging of synaptic vesicle recycling

For measuring recycled synaptic vesicles, neurons were loaded with FM4-64 dye (#T3166, Thermo Fisher) and stimulated with high K^+^- solution, following the previous protocol [19]. Images were acquired before and after destaining with high K^+^- solution for 90 s at 2 s intervals using Eclipse Ts2™ fluorescence microscopy (Nikon, Tokyo, Japan) and underwent analysis of destaining kinetics using Image J.

### Synaptic puncta analysis

Synapse identification was performed by assessing the co-localization of pre-synaptic (synaptophysin) and post-synaptic (PSD-95) puncta using the “Synapse Counter” plug-in in ImageJ. The analysis involved segmenting 20 μm sections along the neurites, followed by image processing with the plug-in. Two independent experiments were conducted, analyzing approximately 20 neurons from five coverslips per condition/group. In each independent culture, 20 μm dendritic segments were evaluated for synaptic protein co-localization, with multiple dendritic regions sampled per NSC and hippocampal neuron. PSD-95/synaptophysin-immunoreactive puncta along the dendrites in each photomicrograph were manually counted.

### Experimental design of animal study

Female pregnant ICR mice exposed to corticosterone mimic the maternal stress-induced mouse model since corticosterone is primarily responsive hormone to stress. Then, the hippocampus of pups was mainly used for evaluating glucocorticoid effect on neurogenesis since the hippocampus is closely related to neuropsychiatric and cognitive function in the brain. Female pregnant ICR mice aged 8 weeks were used, in compliance and approval with the Institutional Animal Care and Use Committee of Seoul National University (SNU-230522-3 and SNU-250221-3). Animals were kept under standard environmental conditions (22 ℃ relative humidity 70%; 12 h light: dark cycle; ad libitum access to food and drinking solution). At least six mice were utilized for each group throughout the study. The experiments were designed in compliance with the ARRIVE guidelines. Allocations of animals were randomly done to minimize the effects of subjective bias. Corticosterone (10 mg/kg), a dose known to saturate the GR for most of the day and reach the serum corticosterone levels comparable to those induced by stress, was dissolved in a solution containing 50% propylene glycol in PBS and administered intraperitoneally daily. This dosage induces maternal stress in female pregnant mice and adult stress in 8-week-old mice [20, 21]. Vehicle-treated mice were injected in the same manner with the solution containing propylene and PBS. After birth, P0 neonates were cryoanesthesized for 2–3 min with crushed ice and the heads were restrained using 3D-printed mouse neonatal stereotaxic adaptor [22]. After the heads were balanced on the AP and ML axes, the needle is moved to (ML, AP) = (-0.8, 1.5 mm) from lambda. Slowly insert the needle until the skin was penetrated, and zero the Z coordinate with the needle at the surface of the skin. We inserted the needle slowly until Z=-1.7 mm and then retracted to -1.5 mm. Viral stocks were diluted to ~ 2 × 10^12^ GC/mL in PBS and FastGreen dye (#F7252, Sigma-Aldrich) was added to a final concentration of 0.1%. Infuse the 2.0 µL viral suspension into the lateral ventricle at the rate of 2.0 µL/minute by Nanoliter2020. We slowly withdraw the needle and repeated for the contralateral hemisphere. Injection efficiency was monitored by the spread of dye throughout the lateral ventricles.

### Behavior tests

After treatment was completed, behavior tests were conducted in a sequence designed to minimize stress and potential confounding effects on performance. Tests began with less stressful tasks and progressed to more stressful ones, as follows: Open field test, Y-maze alternation test, and then forced swim test. Each test was conducted once per day with a 24 h recovery interval between tests to allow the mice to recover from any potential stress induced by the previous test.

Open field test evaluates anxiety of rodents using their characteristics to explore the periphery of open field when anxious. Before the performance, the animals were habituated to the testing room for 3 h to minimize the stress. Mice were placed in the rectangular black plastic boxes (H30 × L30 × W30 cm) and activity was recorded for 10 min. Total distance explored and time spent in either center or periphery of the open field were analyzed using Smart 3.0 video tracking system.

Y-maze spontaneous alternation test depends on the innate instinct of rodents to differently explore new environments. Thus, this test is widely used for quantifying the spatial memory of rodents. Rodents often prefer to challenge a new arm of the Y-maze rather than returning back to the one previously explored. Before the performance, the animals were habituated to the testing room for 3 h to minimize the stress. Then, mice were allowed for 10 min to explore the Y-shaped maze purchased from Sam-Jung Company (Seoul, Korea) while the number of arm entries and triads was recorded to calculate the percentage of an alternation. Only an entry when all four limbs were within the arm was counted. The alternation value represents the number of alternations which was divided by the number of total triads, which equals the number of total entries-2.

Forced swim test is a behavior test for rodents to evaluate depressive-like behavior. Before the performance, the animals were habituated to the testing room for 3 h to minimize the stress. Mice were subjected to a forced swim test for 6 min in a beaker (10 cm × 20 cm) filled with tap water at room temperature, and the trials were analyzed by Smart 3.0 video tracking system (developed by Panlab, Barcelona, Spain). Generally, only the last 4 min of the test is analyzed, because most mice rigorously try to escape the environment at the beginning of the test. Immobility was defined as floating or remaining motionless without leaning against the wall of the cylinder.

### Immunohistochemistry (IHC)

Mice were subjected to deep anesthesia with zoletil (50 mg/ kg) and perfused transcardially with calcium-free Tyrode’s solution, followed by 4% paraformaldehyde. The brains were post-fixed for 2 h in 4% paraformaldehyde and then dehydrated in 30% sucrose in PBS for 24 h at 4 ℃. Serial transverse sections (40 μm) were conducted using a cryostat (Leica Biosystems, Nussloch, Germany). Free-floating method was used to stain the hippocampus. Sections were incubated with 5% NGS dissolved in 1% TritonX-100 at RT for 1 h, and then for 2 d at 4 °C with the primary antibody (1:1,000 dilution). The primary antibodies used in immunocytochemistry were listed as follows: Synaptophysin (#ab32127, Abcam), PSD95 (#MAB1596, Sigma Chemical Company), PSD95 (#51-6900, Thermo fisher), and TOMM20 (#ab56783, Abcam). Samples were incubated with the secondary antibody (1:300 dilution) for 2 h at RT following three-times PBS wash. A confocal microscope system (Carl Zeiss, LSM710) and a super-resolution radial fluctuations (SRRF) imaging system (Andor Technology) were used to visualize the sections. Image analysis was performed using Fiji software.

### Statistical analysis

Imaging experiments and animal tests were conducted and assessed in a blinded fashion. Sample sizes were kept similar between experimental groups and replicates of experiments. The sample size ‘n’ represents the number of biological independent replicates, at least three times and statistical analyses were conducted using these independent values. The Shapiro–Wilk test was used to verify the normality of all data, which were then analyzed using parametric statistics. The unpaired Student’s t test was performed to compare the means of the treatment groups with that of the control group. One-way ANOVA (with Dunnett’s multiple comparison test) or two-way ANOVA (with Tukey’s multiple comparison test) was used for analyzing the differences among multiple groups. For measuring co-localization levels in images, the values of Pearson’s correlation coefficient were obtained from images of each treatment group, and appropriate tests were applied to confirm whether changes were statistically significant. Results were expressed as mean value ± standard error of the mean (S.E.M.) and analyzed with the GraphPad Prism 10 software (GraphPad, CA, USA). A result with a p value of < 0.05 was considered statistically significant.

## Results

### Effects of prenatal glucocorticoid exposure on mitochondrial transport and fusion/fission in differentiating neurons

**Fig. 1:**
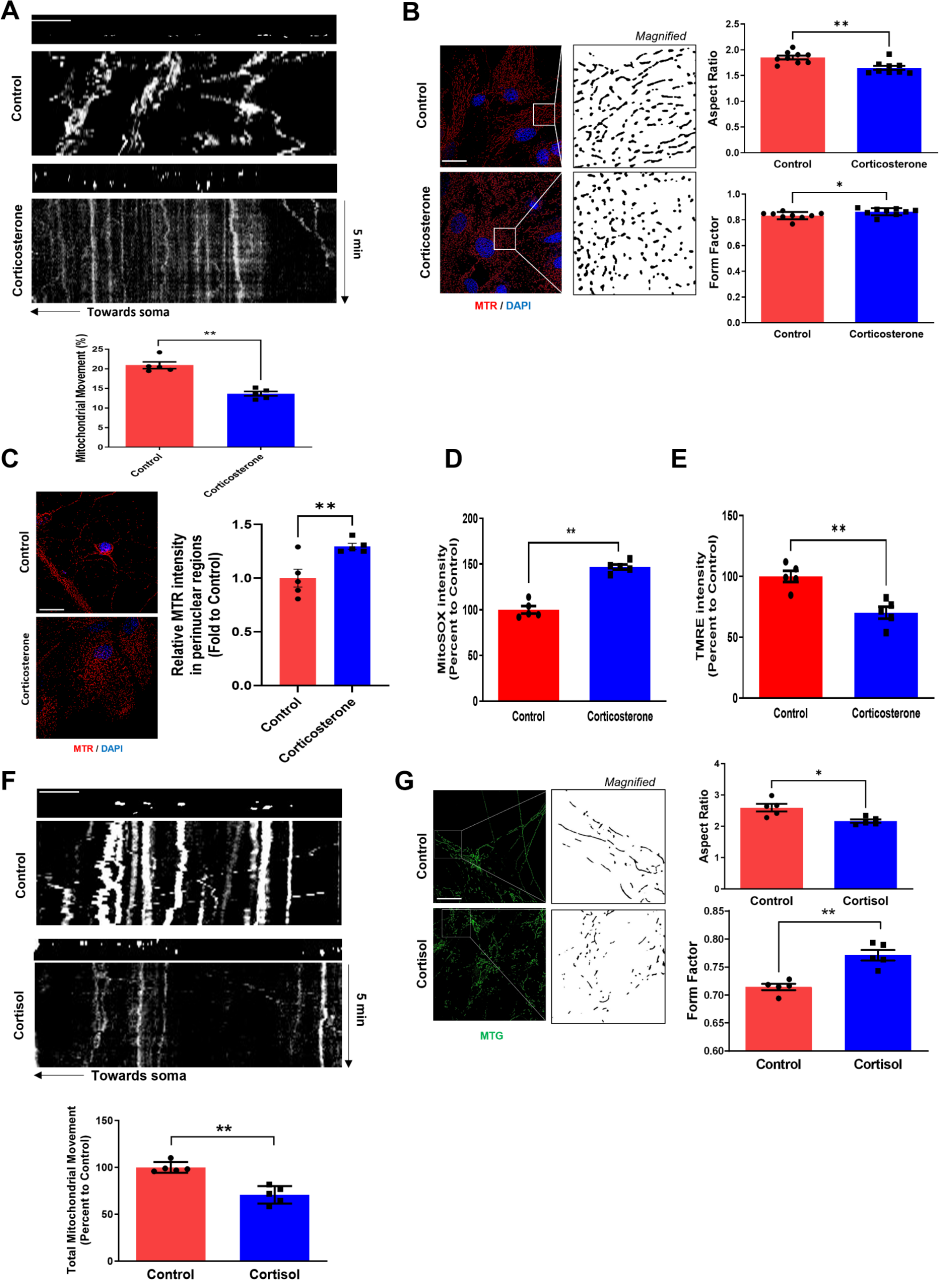
Effects of prenatal glucocorticoid exposure on mitochondrial dynamics in differentiating neurons. (**A**– **E**). Female pregnant mice were exposed to either vehicle or corticosterone (10 mg/kg) at E14 and mouse hippocampal neurons from E18 fetus were cultured until DIV14. (**A**) Hippocampal neurons were stained with MTR (red) and time-lapse imaging of mitochondrial movement of DIV14 neurons were recorded for 5 min. A kymograph was constructed to project time series of mitochondria along the time axis. Scale bars, 10 μm. *n* = 5. (**B**) Hippocampal neurons were immunostained with MitoTracker Red (MTR, red). Quantification of mitochondrial shape descriptors (form factor and aspect ratio) were determined by using Fiji software. Scale bars, 20 μm. *n* = 9. (**C**) Hippocampal neurons were immunostained with MTR (red) and DAPI (blue). Mitochondrial intensity around perinuclear region (nucleus to 20 μm) was quantified by image J. Scale bars, 20 µ. *n* = 5. (**D**) The level of mtROS of hippocampal neurons was measured via MitoSOX staining with luminometer. *n* = 5. (**E**) Mitochondrial membrane potential of hippocampal neurons was measured via tetramethylrhodamine ethyl ester (TMRE) staining with luminometer. *n* = 5. (**F**– **G**) NSCs derived from hiPSC were cultured in neuronal differentiation media for 6 days and treated with cortisol (1 µM) for 48 h. (**F**) Human NSCs were immunostained with MTR (red) and time-lapse imaging of mitochondrial movement were recorded for 5 min. The first frame of the time-lapse image series is shown above of a kymograph generated form the movie. The x-axis corresponds to mitochondrial position and y-axis corresponds to time (progressing from top to bottom). Quantification of mitochondrial mobility in situ and velocity of moving mitochondria in the 5 min period using Fiji software. Scale bars, 10 μm. *n* = 5. (**G**) Human NSCs were immunostained by MTG (green). Quantification of mitochondrial shape descriptors (form factor and aspect ratio) were determined by using Fiji software. Scale bars, 20 μm. *n* = 5. Quantitative data are presented as a mean ± S.E.M. *, ** indicates *p* < 0.05, *p* < 0.01 respectively

We exposed pregnant female mice to corticosterone at E14 to examine the relationship between prenatal stress exposure and mitochondrial transport/morphology in mouse hippocampal neurons at DIV 14. Furthermore, we used hiPSC-derived neurons exposed to high cortisol levels on day 8 post-differentiation because cell growth cones and the number of branches peak at day 9 decreasing afterward [23]. We measured mitochondrial movement as previously reported [24], and mitochondria trafficking kymograph (Supplementary Fig. 1). Prenatal corticosterone significantly reduced mitochondrial motility (Fig. 1A) and caused excessive fission, reducing mitochondrial length (Fig. 1B). Dysfunctional, fragmented mitochondria accumulated around the nucleus, termed perinuclear clumping. Observing MitoTracker Red (MTR) immunofluorescence intensity from the nucleus up to 20 μm, corticosterone increased perinuclear clumping of mitochondria (Fig. 1C). Next, we investigated whether this phenomenon led to mitochondrial dysfunction represented by increased mitochondria-derived ROS and reduced membrane potential. Thus, we stained mouse hippocampal neurons with MitoSOX Red, a cell-permeant mitochondrial superoxide indicator, to measure mitochondrial ROS (mtROS) levels, and tetramethylrhodamine ethyl ester (TMRE) to quantify mitochondrial membrane potential. Prenatal corticosterone exposure increased mtROS and reduced mitochondrial membrane potential (Fig. 1D, E). Similar mitochondrial arrest and excessive fission were observed in hiPSC-derived neurons exposed to cortisol (Fig. 1F, G). Collectively, prenatal glucocorticoid exposure leads to mitochondrial dysfunction in differentiating neurons, characterized by arrest and fragmentation.

In differentiating neurons, extensive mitochondrial trafficking from the cell body toward axons or dendrites contributes to supplying energy for synapse formation and neurotransmission. We hypothesized that prenatal glucocorticoid exposure would reduce mitochondrial distribution around the synapse and subsequent synaptic density during differentiation in neurons. Hippocampal neurons exposed to prenatal glucocorticoid and hiPSC-derived neurons exposed to cortisol did not have significant effect on neuronal cell death (Supplementary Fig. 2A, B). Colocalization studies of mitochondria with synaptophysin and PSD95, marker proteins for the presynaptic and postsynaptic compartments, respectively, revealed significantly reduced mitochondrial presence at dendrites/axons and suppressed synapse formation in hippocampal neurons exposed to corticosterone (Supplementary Fig. 2C, D). Furthermore, prenatal corticosterone exposure repressed the rate of FM4-64 dye release, indicating synaptic vesicle recycling and subsequent synaptic currents were decreased (Supplementary Fig. 2E). Similar reductions in synaptic density and mitochondrial distribution in synaptic regions were observed in hiPSC-derived neurons (Supplementary Fig. 2F, G). These disruptions also led to decreased levels of the synaptic markers PSD95 and synaptophysin. (Supplementary Fig. 2H).

### Prenatal glucocorticoid-induced Miro1 downregulation triggers mitochondrial arrest and excessive fission

**Fig. 2:**
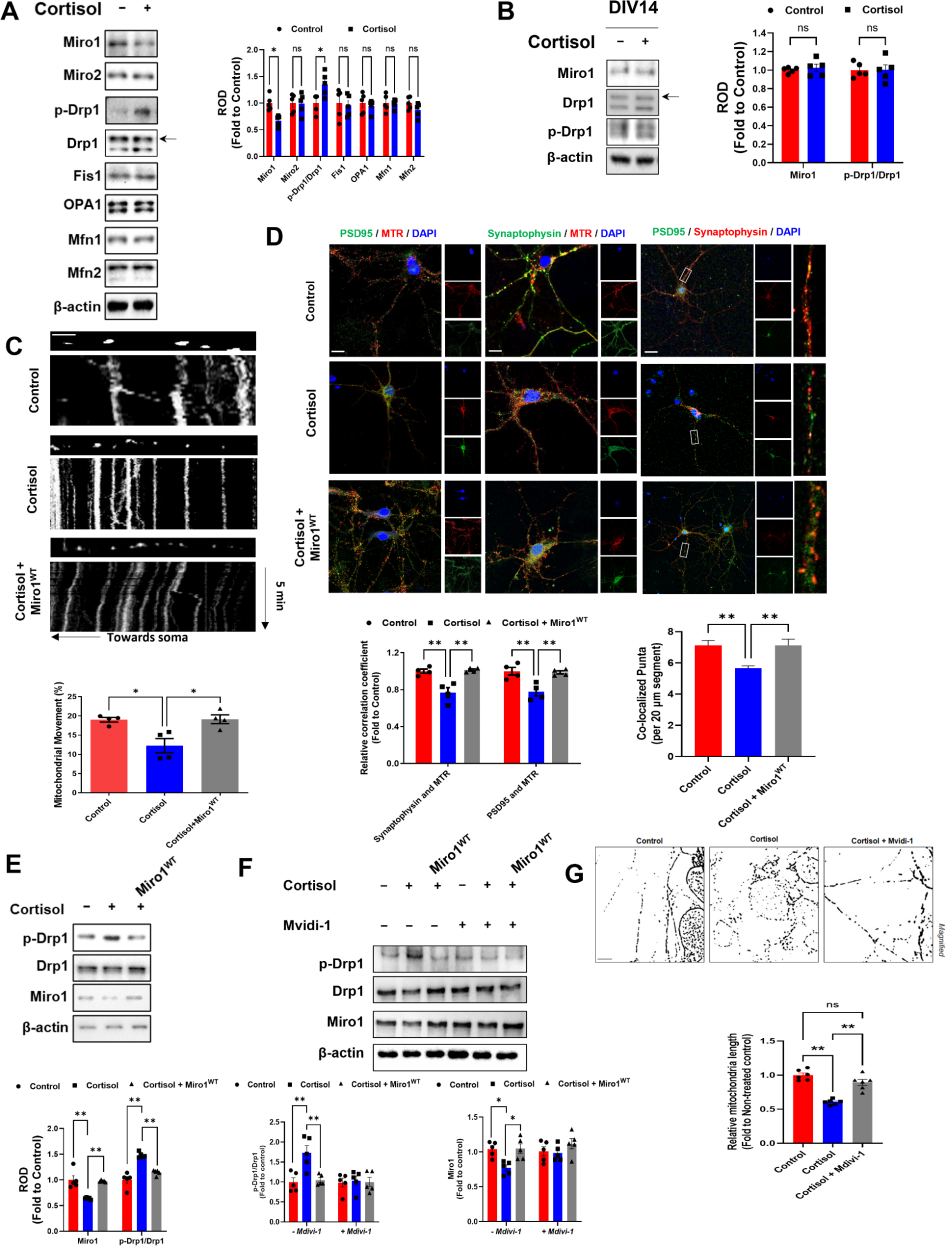
Prenatal glucocorticoid induced dysfunctions in mitochondrial distribution at synapse. (**A**) Human NSCs were cultured in neuronal differentiation media for 6 days and treated with cortisol (1 µM) for 24 h. The expressions of Miro1/2, Drp1, p-Drp1 (Ser616), Fis1, OPA1, and Mfn1/2 were determined by western blot. The β-actin was used as a loading control. *n* = 5. (**B**) Human NSCs were cultured in neuronal differentiation media for 12 days and treated with cortisol (1 µM) for 24 h. The expressions of Miro1, Drp1, and p-Drp1 (Ser616) were determined by western blot. The β-actin was used as a loading control. *n* = 5. (**C**) Human NSCs were transduced with Miro1^WT^ expression vector 24 h after seeding and immunostained with MTR and time-lapse imaging of mitochondrial movement of DIV14 neurons were recorded for 5 min. A kymograph was constructed to project time series of mitochondria along the time axis. Scale bars, 10 μm. *n* = 5. (**D**) Human NSCs were immunostained with synaptophysin, PSD95, and MTR. Pearson’s correlation coefficient was quantified for detecting mitochondrial distribution at synapse. And quantification of the number of synaptophysin/PSD95 puncta pairs per 20 μm. Scale bars, 20 μm. The number of neurites extending from the soma of 20 neurons Scale bars, 20 μm. *n* = 5. (**E**) Human NSCs were cultured in neuronal differentiation media for 6 days and treated with cortisol (1 µM) for 24 h. The expressions of Miro1, Drp1, and p-Drp1 (Ser616) were determined by western blot. The β-actin was used as a loading control. *n* = 5. (**F**) Human NSCs were cultured in neuronal differentiation media for 6 days and treated with Mvidi-1 (10 µM) for 30 min prior to cortisol treatment. The expressions of Miro1, Drp1, and p-Drp1 were determined by western blot. The β-actin was used as a loading control. *n* = 5. (**G**) Cortisol mediated mitochondrial fission inhibited by Mvidi-1. *n* = 6. Quantitative data are presented as a mean ± S.E.M. The representative images were acquired by confocal microscope system and SRRF imaging system. *, ** indicates *p* < 0.05, *p* < 0.01 respectively. ns means not significant

Mitochondrial dynamics-related proteins include mitochondrial trafficking proteins such as Miro1/2 and mitochondrial morphology regulating proteins including fusion proteins (OPA1, Mfn1/2) and fission proteins (Drp1, Fis1). We investigated which regulators of mitochondrial dynamics changed upon cortisol during neurogenesis. We identified Miro1 downregulation and increased Drp1 phosphorylation (Ser616) as key changes during neurogenesis, without significant changes in other factors (Fig. 2A). This effect was specific to differentiating neurons while terminally differentiated neurons (DIV 14) did not show these changes (Fig. 2B). We also found that Miro1 downregulation and increased Drp1 phosphorylation (Ser616) during neurogenesis, without significant changes in other mitochondrial dynamics proteins (Miro2, Fis1, OPA1, Mfn1/2) and Drp1 phosphorylation (Ser616) related kinases (ERK, CAMKII, Cdk5) in corticosterone exposed mouse hippocampal neurons at DIV 14 (Supplementary Fig. 3A, B).

It has been widely known that mitochondrial transport is mainly mediated by Miro1/2 protein interacting with adaptor proteins such as kinesin or dynein to move mitochondria along cytoskeleton track [25]. Miro tethers mitochondria towards synapse with the help of motor protein kinesin 1. Miro1 downregulation disrupted the formation of mitochondria-Miro1-kinesin 1 complex, essential for mitochondrial transport, reducing interactions between kinesin 1 and mitochondria, and between Miro1 and mitochondria in mouse hippocampal neurons (Supplementary Fig. 3C). Similarly, the interaction between kinesin 1 and Miro1, and between kinesin 1 and α-tubulin, was reduced in hiPSC-derived neurons exposed to cortisol, where Miro1 downregulation triggered the derailment of mitochondria from microtubules (Supplementary Fig. 3D, E).

To determine whether a reduction in Miro1 is involved in cortisol-induced mitochondrial arrest, we investigated whether Miro1 overexpression had a restorative effect. With live cell imaging, we observed that the Miro1^WT^ expression restored mitochondrial motility (Fig. 2C), recovered interaction with kinesin 1 and α-tubulin (Supplementary Fig. 3F), and normalized mitochondrial distribution at synaptic regions (Fig. 2D, Supplementary Fig. 3G).

To elucidate the hierarchical relationship between Miro1 and Drp1 in regulating mitochondrial dynamics, we conducted a series of experiments to identify which protein acts as the upstream regulator. Our approach involved examining the effects of Miro1 overexpression and Drp1 inhibition on each other’s expression and activity. Initially, we overexpressed Miro1 in differentiating neurons and assessed the subsequent levels and phosphorylation state of Drp1. The results showed that Miro1 upregulation significantly reduced Drp1 phosphorylation (Ser616) to control levels, indicating that Miro1 can reduce the phosphorylation of Drp1, which could result in decreased mitochondrial fission (Fig. 2E). Additionally, we inhibited Drp1 activity using mdivi-1. This inhibition did not result in any significant changes in the protein levels of Miro1 (Fig. 2F). Furthermore, Drp1 overexpression had no effect on the expression of Miro1, and Mdivi-1 treatment inhibited Drp1-mediated mitochondrial fission suggesting that Drp1 does not exert upstream control over Miro1 (Fig. 2G, Supplementary Fig. 3H). Also, this inhibition did not have a significant effect on neuronal cell death (Supplementary Fig. 3I). Furthermore, Miro1 silencing increases mitochondria fission and Drp1 activity (Supplementary Fig. 4). This result reinforces the notion that Miro1 functions as an upstream regulator of Drp1 activity. Our findings indicate that Miro1 is the upstream regulator influencing Drp1 activity, rather than the other way around. Based on these results, we hypothesized that Miro1 controls mitochondrial arrest as well as excessive fission through its regulation of Drp1 phosphorylation (Ser616) and activity.

### Effects N-terminal GTPase activity reduction of Miro1 by cortisol in ERMC formation and subsequent Drp1 activation in differentiating neurons

Next, the role of Miro1 in mitochondrial shape transition (MiST) by Drp1 regulation was investigated in neurons exposed to prenatal glucocorticoid. Recently, Miro1 has been suggested to participate in ER-mitochondrial connection (ERMC)-dependent MiST as Miro1 and Drp1 activity is significantly dependent on Ca^2+^ levels [26]. There is controversy as to whether domains of the Miro1 protein regulate its Ca^2+^ binding property or concentration control. While some suggest that the EF-hand I/II domain of Miro1 binds to Ca^2+^ and regulates mitochondrial distribution, others demonstrated that the Rho GTPase domain is involved in mitochondrial distribution to facilitate the interaction of mitochondria with kinesin/dynein or TRAK protein [27]. However, it is unclear which domains of Miro1 are involved in ERMC-dependent MiST.

**Fig. 3:**
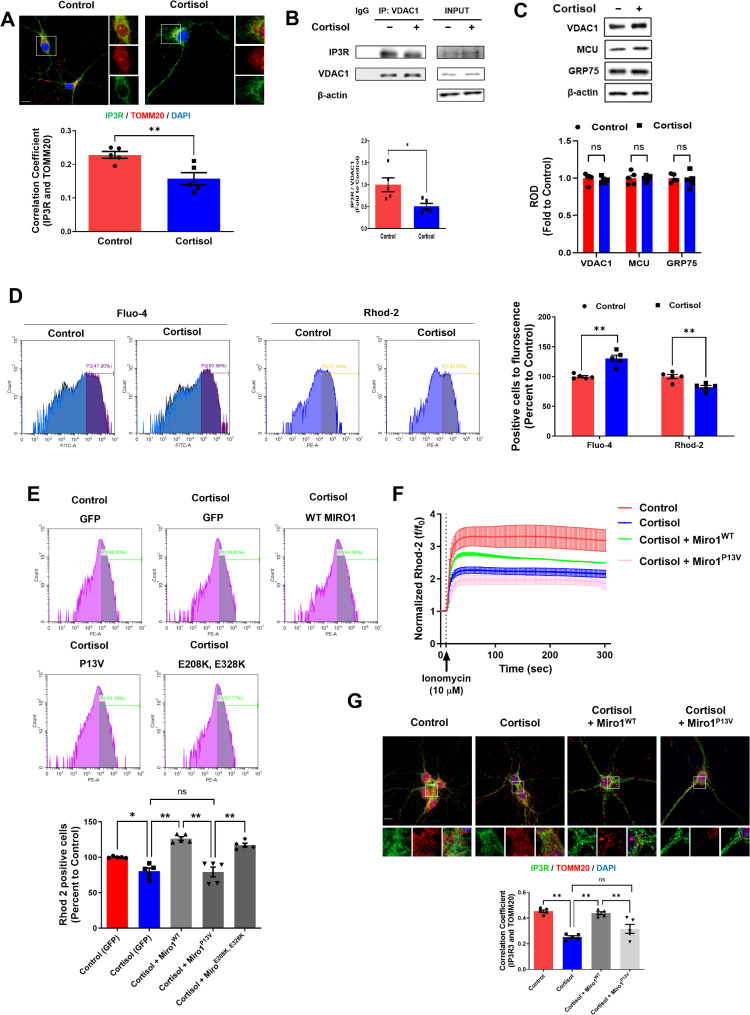
Effects of Miro1 downregulation by cortisol on ERMC tethering and intra-mitochondrial Ca^2+^ levels. (**A**– **G**) Human NSCs were cultured in neuronal differentiation media for 6 days and treated with cortisol (1 µM) for 24 h. (**A**) Human NSCs were immunostained with IP3R, TOMM20, and DAPI. Pearson’s correlation coefficient was quantified. Scale bars, 10 μm. *n =* 5. (**B**) VDAC1 was co-immunoprecipitated with IP3R. IP3R levels in immunoprecipitated samples were quantified. *n =* 5. (**C**) The expressions of VDAC1, MCU, and GRP75 were determined by western blot. The β-actin was used as a loading control. *n =* 5. (**D**) Human NSCs were treated with Rhod-2 (3 µM) and Fluo-4 (3 µM) for 1 h prior to evaluating mitochondrial Ca^2+^ and cytoplasmic Ca^2+^ level, respectively. *n =* 5. (**E**– **G**) Human NSCs were transduced with Miro1^WT^, Miro1^P13V^, and Miro1^E208K, E328K^ expression vector 24 h after seeding. (**E**) Cells were treated with Rhod-2 for 1 h prior to detection of mitochondrial Ca^2+^ level. Rhod-2 positive cells were quantified by FACS. (**F**) Cells were treated with Rhod-2 for 1 h prior to detection of mitochondrial Ca^2+^ level. Time-lapse imaging was done over 300 s at 2 s intervals while cells were treated with calcium ionophore ionomycin (10 µM) at 10 s. *n =* 5. (**G**) Human NSCs were immunostained with IP3R, TOMM20, and DAPI. Pearson’s correlation coefficient was quantified. Scale bars, 10 μm. *n =* 5. Quantitative data are presented as a mean ± S.E.M. The representative images were acquired by confocal microscope system. *, ** indicates *p* < 0.05, *p* < 0.01 respectively. ns means not significant

We then first observed ERMC formation through observing the interaction between mitochondria-associated membrane of ER (MAM) protein IP3R and mitochondrial protein TOMM20 or VDAC1. ERMC formation, represented by IP3R-TOMM20/VDAC1 binding, was significantly reduced by cortisol in iPSC-derived neurons (Fig. 3A, B). However, the expression of ERMC-related proteins such as IP3R, VDAC1, MCU, and GPR75 remained unchanged (Fig. 3C). ERMC formation triggers calcium transfer from ER to mitochondria. We then measured cytosolic and mitochondrial Ca^2+^ levels in differentiating neurons through Fluo-4 and Rhod-2 detection, respectively. Cortisol increased cytosolic Ca^2+^ but reduced mitochondrial Ca^2+^, indicating that the reduction in ERMC decreased mitochondrial Ca^2+^ influx from the ER (Fig. 3D). We then performed a mutagenesis study to identify the domain associated with this phenomenon by expressing a Miro1 bearing a constitutively active N-terminal GTPase domain (Miro1^P13V^) and defects in EF-hand I/II (Miro1^E208K, E328K^; see Supplementary Fig. 5). The P13V mutation was predicted to lock the N-terminal GTPase in the GTP-bound state, modestly decreasing the affinity of Miro1 for TRAK. Interestingly, we found that a mutation in the N-terminal GTPase of Miro1 has a critical role in mitochondrial Ca^2+^ influx, unlike many previous studies suggest that only EF hands of Miro1 regulate Ca^2+^ dynamics (Fig. 3E).

The GTPase domain of Miro1 is known to recruit the heavy chain of kinesin and motor adaptor protein Milton/Trak1, but until recently, the effect of this domain on mitochondrial dysfunction remained elusive [26]. Here, we found that the N-terminal GTPase domain of Miro1 is highly associated with mitochondrial calcium dynamics. Mitochondrial Ca^2+^ influx was reduced by defects in the N-terminal GTPase activity of Miro1 due to cortisol even though the cytoplasmic Ca^2+^ level was artificially increased with ionomycin (Fig. 3F). Miro1 restoration recovered ERMC formation, whereas Miro1^P13V^ overexpression reduced its formation (Fig. 3G). These findings highlight the crucial role of the N-terminal GTPase function of Miro1 in maintaining ERMC formation, which is suppressed by glucocorticoid treatment during differentiation. Several studies demonstrated that Miro1 can increase the amount of mitophagy, which can also contribute to Drp1-mediated mitochondrial fragmentation. We detected colocalization between the lysosome marker LAMP1 and a mitochondrial marker but no significant changes in LAMP1 and MTR colocalization in hippocampal neurons were detected, indicating that Miro1 is not associated with mitophagy-related MiST (Supplementary Fig. 6A). Additionally, the autophagy-related marker LC3 has no significant change in hippocampal neurons (Supplementary Fig. 6B).

**Fig. 4:**
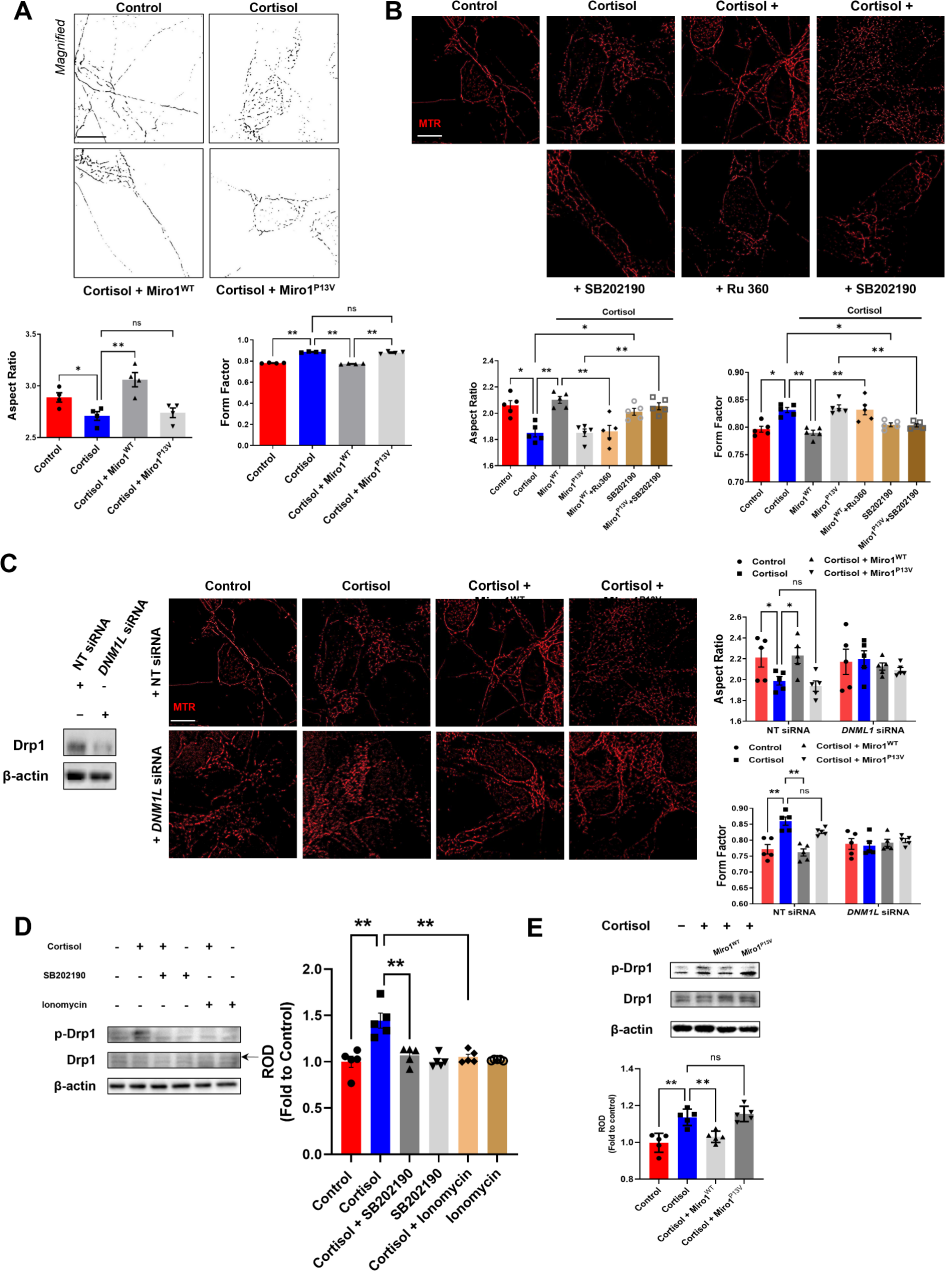
Reduced intra-mitochondrial Ca^2+^contributed to Drp1-mediated mitochondrial fission. (**A**, **C**, **E**) Human NSCs were cultured in neuronal differentiation media for 6 days and treated with cortisol (1 µM) for 24 h. Furthermore, human NSCs were transduced with control vector, Miro1^WT^, or Miro1^P13V^ expression vector 24 h after seeding. (**A**– **C**) Human NSCs were immunostained with mitotracker Red (MTR, red). (**A**) Quantification of mitochondrial shape descriptors (form factor and aspect ratio) were determined by using Fiji software. Scale bars, 20 μm. *n =* 4. (**B**) Human NSCs were treated with SB202190 (10 µM) or Ru 360 (10 µM) 30 min before cortisol treatment. *n =* 5. (**C**) Human NSCs were transfected with NT siRNA or *DNM1L* siRNA 24 h before cortisol treatment. *n =* 5. The expressions of Drp1 were determined by western blot. The β-actin was used as a loading control. (**D**) Human NSCs were cultured in neuronal differentiation media for 6 days and treated with SB 202,190 or ionomycin (10 µM) for 30 min prior to cortisol treatment. The expressions of p-Drp1 (Ser616) and Drp1 were determined by western blot. The β-actin was used as a loading control. *n =* 5. (**E**) The expressions of p-Drp1 and Drp1 were determined by western blot. The β-actin was used as a loading control. *n =* 5. Quantitative data are presented as a mean ± S.E.M. The representative images were acquired by SRRF imaging system. *, ** indicates *p* < 0.05, *p* < 0.01 respectively. ns means not significant

The exact mechanism whereby mitochondrial transport affects mitochondrial fusion/fission remains elusive [28]. Our results suggest that intracellular Ca^2+^-dependent Drp1 activation may be associated with reduced ERMC formation by Miro1 downregulation, highlighting the role of Ca^2+^ signaling in mitochondrial dynamics. Based on this, we hypothesized that the N-terminal GTPase domain of Miro1 serves as a key regulatory element in promoting mitochondrial fission by modulating Drp1 activity. First, we confirmed that Miro1^WT^ transfection alleviated excessive mitochondrial fission by cortisol, while Miro1^P13V^ expression did not prevent this effect (Fig. 4A). To further explore the role of mitochondrial Ca^2+^ levels in fragmentation, we treated cells with Ru360 and SB202190, which suppresses and activates mitochondrial Ca^2+^ influx, respectively. Excessive mitochondrial fission by either cortisol or cortisol with Miro1^P13V^ overexpression, both of which induce reduction in mitochondrial Ca^2+^ levels, was reversed by SB202190, suggesting that maintaining mitochondrial Ca^2+^ is crucial for preserving mitochondrial morphology. Conversely, restored mitochondrial morphology observed with cortisol and the overexpression of Miro1^WT^ was disrupted by Ru 360, reinforcing the significance of mitochondrial Ca^2+^ in Drp1 activation (Fig. 4B). This aligns with findings that Miro1 knockdown, as an upstream regulator of Drp1 activity, leads to deficient mitochondrial Ca^2+^ and cell death [24]. Then, the increased Drp1 activity will trigger mitochondrial fission. We therefore hypothesized that downregulating this protein would reverse the increasing effect of Miro1 reduction. As expected, *DNM1L* knockdown suppressed the mitochondrial fission triggered by cortisol and Miro1^P13V^ overexpression (Fig. 4C). We observed that cortisol increased Drp1 phosphorylation (Ser616), which was mitigated by increasing mitochondrial Ca^2+^ influx with SB202190 and ionomycin (Fig. 4D). Finally, we determined that Miro1 upregulation reduced cortisol-induced Drp1 activity, whereas Miro1^P13V^ did not affect the phosphorylation state of Drp1 (Fig. 4E).

### Recovery of Miro1 N-terminal GTPase function mitigates glucocorticoid-induced mitochondrial and synaptic dysfunction

**Fig. 5:**
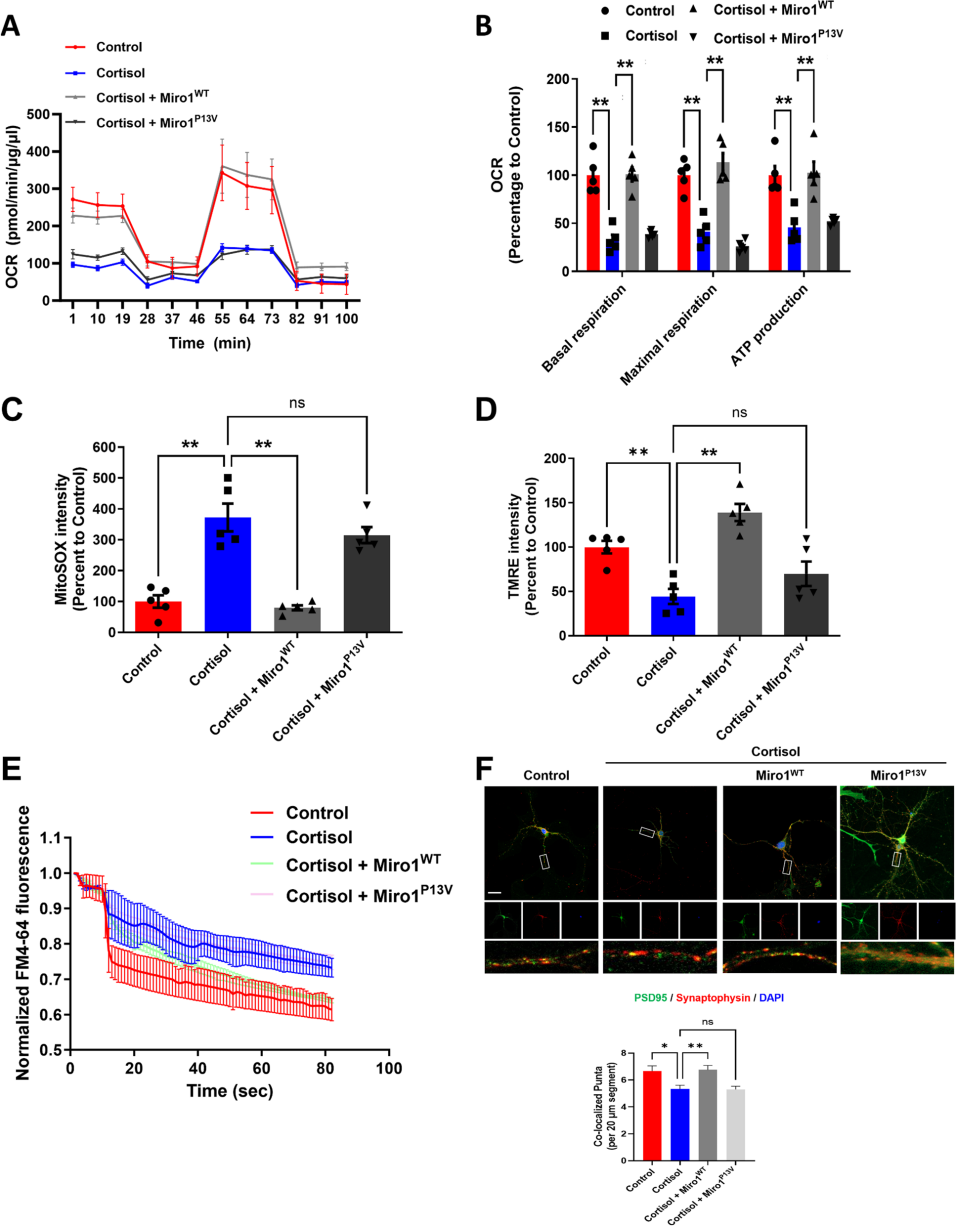
Restoration of Miro1 N-terminal GTPase recovered the deleterious effects of prenatal cortisol on mitochondrial function and synaptogenesis. (**A**– **F**) Human NSCs were cultured in neuronal differentiation media for 6 days and treated with cortisol (1 µM) for 48 h. Furthermore, human NSCs were transduced with control vector, Miro1^WT^, or Miro1^P13V^ expression vector 24 h after seeding. (**A**– **B**) Oxygen consumption rate (OCR) changes under mitochondrial stress test were measured by using Seahorse SF24 Extracellular Flux analyzer where oligomycin, carbonyl cyanide-4-(trifluoromethoxy)phenylhydrazone (FCCP), and antimycin A/rotenone mixture were treated. Statistics of basal respiration, maximal respiration, and ATP production were also presented. *n* = 5. (**C**) The levels of mtROS were measured via MitoSOX staining with luminometer. *n* = 5. (**D**) Mitochondrial membrane potential was measured via TMRE staining with luminometer. *n* = 5. (**E**) Human NSCs were stained with FM4-64 dye and stimulated with high K^+^ buffer for destaining. Time-lapse imaging was done over 90 s at 1 s intervals with an Eclipse Ts2TM fluorescence microscopy. *n =* 5. (**F**) Human NSCs were immunostained with PSD95, synaptophysin, and DAPI. Quantification of the number of synaptophysin/PSD95 puncta pairs per 20 μm. Scale bars, 20 μm. The number of neurites extending from the soma of 20 neurons. Scale bars, 10 μm. *n =* 5. Quantitative data are presented as a mean ± S.E.M. The representative images were acquired by confocal microscope system. ** indicates *p* < 0.01. ns means not significant

Excessive mitochondrial fission, as well as their arrest, contribute to suppress mitochondrial function, resulting in energy deficiency and subsequent synapse dysfunction. Thus, we investigated whether Miro1 upregulation could recover mitochondrial and synaptic function but impaired by defects in its N-terminal GTPase domain. Oxygen consumption rate data showed that cortisol decreased basal respiration, maximal respiration, and ATP production. These effects were ameliorated by Miro1 overexpression. However, Miro1^P13V^ expression did not reverse the effects of cortisol on these parameters (Fig. 5A, B). The increased mtROS and decreased mitochondrial membrane potential caused by cortisol were reversed by Miro1^WT^ expression, but not with Miro1^P13V^ expression (Fig. 5C, D). With mitochondria function recovery, synapse formation and neurotransmission will become normal as synapse uses up tremendous ATP. Synaptic vesicle recycling and density were also normalized by Miro1^WT^ expression, but not by Miro1^P13V^ expression (Fig. 5E, F). In summary, the N-terminal GTPase of Miro1 appears to be a master regulator of Drp1-mediated fission in differentiating neurons exposed to prenatal glucocorticoids.

**Fig. 6:**
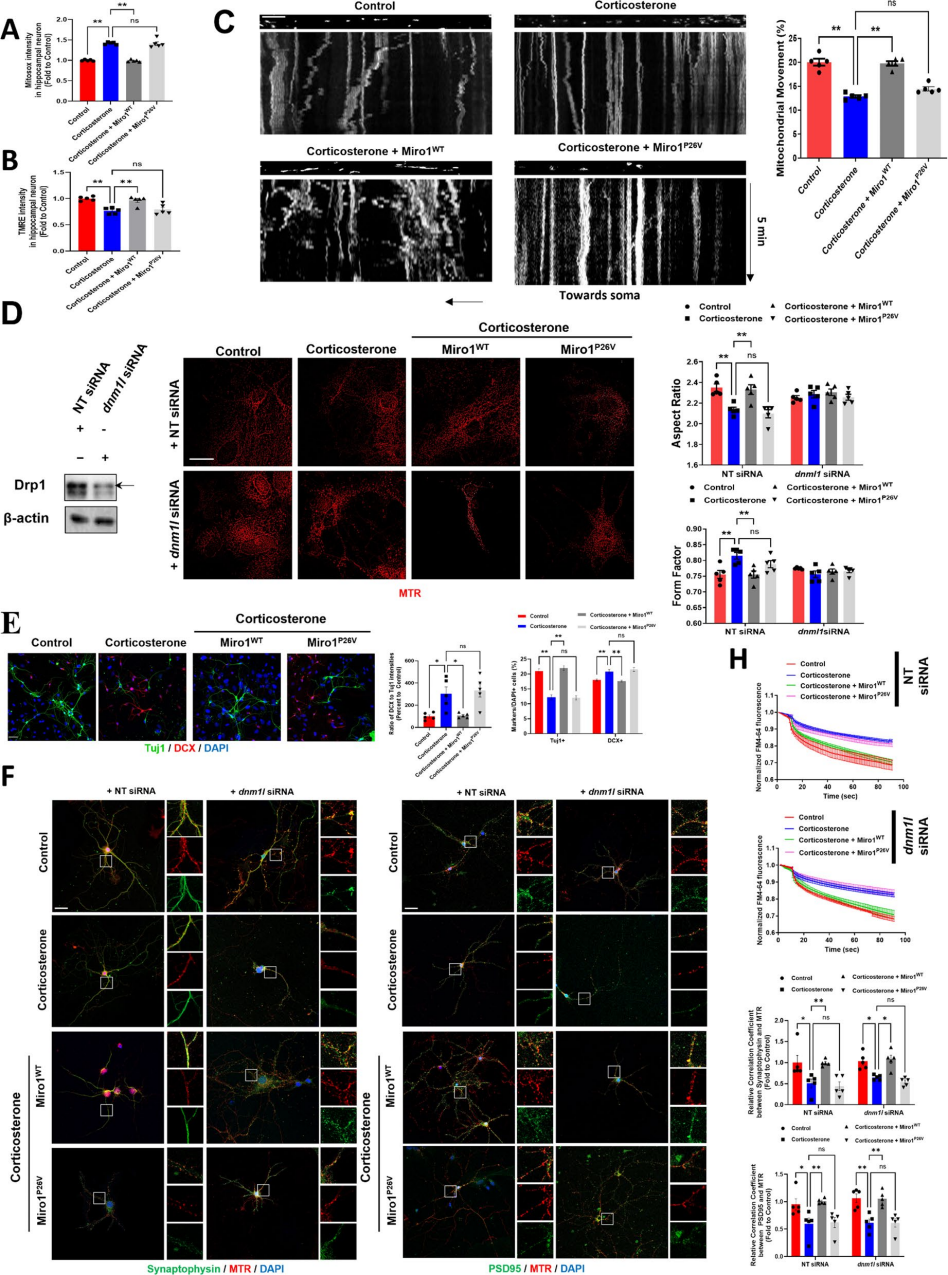
Restoration of Miro1 N-terminal GTPase recovered prenatal corticosterone-induced dysfunctions in mitochondrial dynamics, mitochondria, and synaptogenesis. (**A**– **F**). Female pregnant mice were exposed to either vehicle or corticosterone (10 mg/kg) at E14 and mouse hippocampal neurons from E18 fetus were cultured until DIV14. Furthermore, hippocampal neurons were transduced with control vector, Miro1^WT^, or Miro1^P26V^ expression vector at DIV1. (**A**) The levels of mtROS of hippocampal neurons were measured via MitoSOX staining with luminometer. *n* = 5. (**B**) Mitochondrial membrane potential of hippocampal neurons was measured via TMRE staining with luminometer. *n* = 5. (**C**) Hippocampal neurons were transduced with control vector, Miro1^WT^, or Miro1^P26V^ expression vector at DIV1 and stained with MTR (red) and time-lapse imaging of mitochondrial movement of DIV14 neurons were recorded for 5 min. A kymograph was constructed to project time series of mitochondria along the time axis. Scale bars, 10 μm (magnification, x 630). *n* = 5. (**D**, **F**– **H**) Nontargeting (NT) or *dnmt1l* siRNA was transfected 48 h before imaging. The expressions of Drp1 were determined by western blot. The β-actin was used as a loading control. (**D**) Hippocampal neurons were immunostained with MTR (red). Quantification of mitochondrial shape descriptors (form factor and aspect ratio) were determined by using Fiji software. Scale bars, 20 μm (magnification, x 1,000). *n =* 5. (**E**) Hippocampal neurons were immunostained with Tuj1, DCX, and DAPI and quantified by calculating the ratio between Tuj1+/DCX + cells and total number of cells. Scale bars, 20 μm. *n* = 30 per group. (**F**– **G**). Hippocampal neurons were immunostained with synaptophysin/PSD95, MTR, and DAPI. GFP represents control or Miro1. Pearson’s correlation coefficient was determined by Fiji1 software. Scale bars, 20 μm. *n =* 5. (**H**) Hippocampal neurons were stained with FM4-64 dye and stimulated with high K^+^ buffer for destaining. Time-lapse imaging was done over 180 s at 1 s intervals with an Eclipse Ts2TM fluorescence microscopy. *n =* 5. Quantitative data are presented as a mean ± S.E.M. The representative images were acquired by confocal microscope system and SRRF imaging system. *, ** indicates *p* < 0.05, *p* < 0.01 respectively. ns means not significant

In addition, we performed in vitro experiments using DIV 14 mouse hippocampal neurons to explore the role of the N-terminal GTPase of Miro1 in mice exposed to prenatal glucocorticoids at E14. Overexpression of Miro1 reversed the increased mtROS and reduced mitochondrial membrane potential induced by prenatal corticosterone exposure. In contrast, expression of Miro1 with a constitutively active N-terminal GTPase domain (Miro1^P26V^) in mice had no significant effect (Fig. 6A, B). The decreased mitochondrial movement due to corticosterone was recovered by Miro1^WT^ expression but not by Miro1^P26V^ transfection (Fig. 6C). Furthermore, *dnm1l* knockdown suppressed mitochondrial fission induced by corticosterone treatment or Miro1^P26V^ transfection (Fig. 6D).

Next, we assessed the impact of Miro1 defects on synaptic function and neurogenesis. To evaluate neurogenesis, we stained hippocampal neurons with doublecortin (DCX, immature neuron marker) and Tuj1 (mature neuron marker). If the ratio of DCX to Tuj1 is high, neurogenesis defects occur with slow differentiation processes. Also, we calculated DCX + and Tuj1 + cells. The ratio of DCX to Tuj1 was increased by corticosterone or Miro1^P26V^ overexpression and returned to control levels with Miro1 overexpression (Fig. 6E). To determine the importance of the N-terminal GTPase of Miro1 in recovering corticosterone-induced synaptic dysfunction, we examined mitochondrial distribution in the pre/postsynaptic compartments. The overexpression of Miro1, but not Miro1^P26V^ expression recovered mitochondrial distribution around synapses (colocalization between mitochondrial and synaptic markers) reduced by corticosterone. However, *dnm1l* knockdown did not dramatically alter this finding, indicating that simply inhibiting excessive mitochondrial fission is insufficient to restore synaptic function without normalizing Miro1 activity (Fig. 6F, G). Similarly, the synaptic dysfunction induced by corticosterone or Miro1^P26V^ expression was not normalized to control levels with Drp1 downregulation alone (Fig. 6H). This underscores the critical role of the N-terminal GTPase domain of Miro1 in regulating synaptic function through its control over Drp1 activity.

### Role of Miro1 N-terminal GTPase in reversing prenatal corticosterone-induced neuropsychiatric and cognitive impairments

**Fig. 7:**
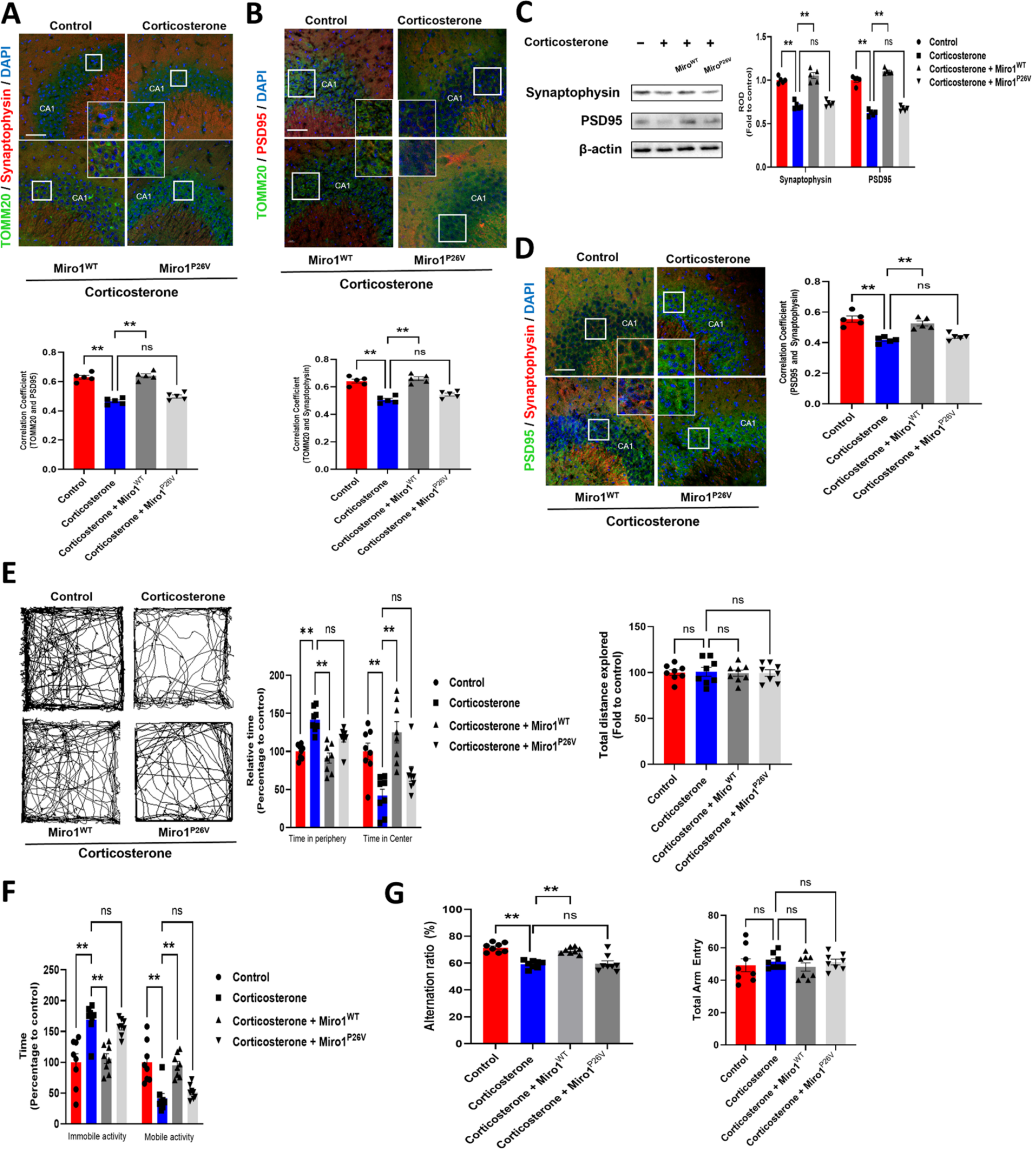
Defects in N-terminal GTPase of Miro1 mainly triggered prenatal corticosterone-induced anxiety/depression-like behavior and spatial memory dysfunction. (**A**– **G**) The AAV vectors (pAAV-GFP-CMV, pAAV-GFP-CMV-Miro1, pAAV-GFP-CMV-Miro1^P26V^) were transduced into lateral ventricle of P0 offspring from female mice exposed to vehicle or corticosterone (10 mg/kg) at E14. P21 mice underwent behavior tests and were sacrificed. (**A**– **B**) Slide samples for IHC were immunostained with TOMM20, synaptophysin/PSD95, and DAPI. GFP represents control or Miro1. Pearson’s correlation coefficient values were quantified by Fiji software. Scale bars, 20 μm. *n =* 5. (**C**) The expression of synaptophysin and PSD95 were determined by western blot where β-actin was used as a loading control. *n =* 5. (**D**) Slide samples for IHC were immunostained with PSD95, synaptophysin, and DAPI. GFP represents control or Miro1. Pearson’s correlation coefficient values were quantified by Fiji software. Scale bars, 100 μm (magnification x 200). *n =* 5. (**E**) Open field test was performed to assess anxiety-like behavior for 10 min. Relative time at the periphery/center and total distance was determined. *n* = 8. (**F**) Mice were presented to the forced swim test for 6 min. Immobile and mobile activities were determined during the last 4 min. *n =* 8. (**G**) The mice were subjected to Y-maze test to evaluate spatial memory function. Alternation ratio and total arm entry was determined. *n =* 8. Quantitative data are presented as a mean ± S.E.M. The representative images were acquired by confocal microscope system. ** indicates *p* < 0.01. ns means not significant

The in vivo experiments confirmed the role of Miro1 in the mouse brain and behavioral changes after exposure to prenatal glucocorticoids. After exposure at E14, GFP-Miro1-AAV transfection was done via intraventricular injection into the P1 neonatal mouse brain (Supplementary Fig. 7A, B). At P21, GFP expression was detected in all mouse brains (Supplementary Fig. 7C). We found that Miro1 downregulation and p-Drp1 upregulation is remained in brain of prenatal stress mouse model (Supplementary Fig. 7D). Also, we confirmed that the reduced distribution of mitochondria at synaptic regions was recovered to control levels following Miro1^WT^ but were not changed by Miro1^P26V^ expression (Fig. 7A, B). The synaptic marker expression and synaptic density represented by colocalization between pre- and post-synaptic markers were normalized by Miro1^WT^ but not by Miro1^P26V^ transfection, (Fig. 7C, D). Previous research showed an association between excessive exposure to corticosterone in fetal rodents and the development of mood disorders or memory impairment in juvenile and adulthood [29, 30]. A forced swimming test was performed to assess depressive-like behavior, by measuring immobility in a cylinder as an indicator of unwillingness to escape the environment. Mice with Miro1^P26V^ expression showed more immobility, whereas Miro1^WT^ expression rescued the depressive mood induced by prenatal corticosterone exposure (Fig. 7E). Using an open field test to observe anxiety behavior (mice with anxiety prefer to stay at the periphery), we confirmed that Miro1^WT^ transfection decreased anxiety behavior, whereas Miro1^P26V^ did not (Fig. 7F). Furthermore, we assessed spatial memory performance with the Y-maze, which exploits rodents’ natural tendency to explore novel objects. Corticosterone-exposed mice showed impaired spatial memory, whereas those with Miro1^WT^ expression exhibited recovered cognition and mice with Miro1^P26V^ expression showed dysfunctional memory (Fig. 7G). Overall, prenatal corticosterone exposure impaired neuropsychiatric and cognitive function in pups, which were restored by Miro1^WT^ but not by Miro1^P26V^. Thus, restoring Miro1, especially the N-terminal GTPase domain, is essential for protecting neural development against prenatal glucocorticoid-induced mitochondrial dysfunction.

## Discussion

**Fig. 8:**
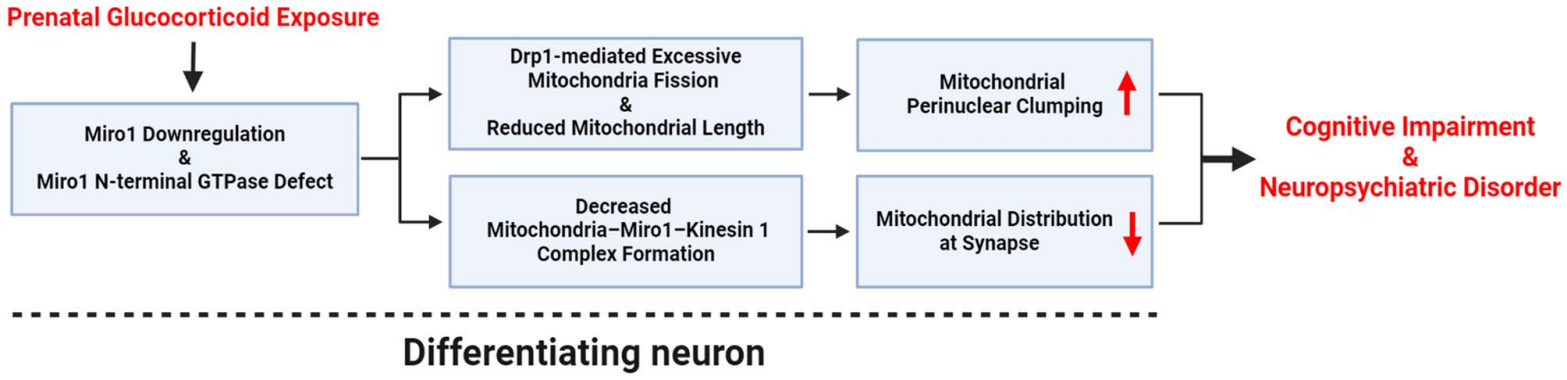
The schematic image for mechanisms of inhibition in Miro1-dependent mitochondrial dynamics by prenatal glucocorticoid

This study provides compelling evidence that prenatal glucocorticoid exposure leads to the downregulation of Miro1, disrupting the mitochondrial fusion/fission cycle in differentiating neurons (Fig. 8). Notably, glucocorticoid-induced defects in the N-terminal GTPase domain of Miro1 act as a pivotal regulator, promoting the activation of Drp1. This complex interaction highlights Miro1’s multifaceted role in mitochondrial dynamics, extending its established function in mitochondrial transport. Prenatal stress can exert long-lasting effects on neuronal homeostasis, often leading to epigenetic and genetic modifications that increase vulnerability to neurological disorders, including Alzheimer's disease (AD) and depression [31–35]. These alterations in brain development and function emphasize the importance of understanding mitochondrial mechanisms involved in prenatal stress-induced neurological disorders.

Mitochondria are highly sensitive to glucocorticoid levels as the organelles responsible for energy production to sustain the stress response. Glucocorticoid has a biphasic effect on neuronal mitochondria, enhancing Ca^2+^ buffering and maintaining mitochondrial membrane potential at physiological levels. In contrast, high levels of glucocorticoid, induced by stress, alter mitochondrial fission/fusion, suppress mitochondrial biogenesis, and promote the accumulation of damaged mitochondria [36]. However, the underlying mechanism can vary depending on the timing of stress exposure. Exposure to prenatal stress induces permanent epigenetic and genetic changes during neurogenesis which continuously affect the brain throughout the lifetime even though stress is discontinued. Unlike stress experienced in adulthood, prenatal stress can thereby have more profound effects due to the brain’s developmental stage, particularly affecting the limbic system, which is more vulnerable to early-life stress because of its later developmental timeline [4, 37, 38]. Mitochondrial dysfunction in this brain region is significantly impacted by glucocorticoid exposure due to the high abundance of glucocorticoid receptors (GRs). In a mouse model of early stress, defects in oxidative phosphorylation of hypothalamic mitochondria, along with hyperactivation of the HPA axis and elevated serum corticosterone, have been observed. These changes alter the expression of genes related to electron transport chain activity and Fis1 protein, contributing to mitochondrial dysfunction [39]. Our and other previous studies also showed that prenatal glucocorticoid exposure reduced mitochondrial numbers around the synapses and impaired mitochondrial function, leading to defects in biogenesis, neurogenesis, and neurodegenerative changes [36, 40, 41]. However, the precise molecular mechanism driving these mitochondrial dysfunctions under prenatal stress remained unclear.

Our study identifies Miro1 as a core protein mediating mitochondrial dysfunction, encompassing not only mitochondrial trafficking but also morphology under prenatal stress conditions. Miro 1, known primarily for its role in mitochondrial transport, tethers mitochondria to microtubules, facilitating axonal transport. Downregulation of Miro1 disrupts mitochondrial transport and predisposes neurons to aging and neurological disorders [5]. In line with previous findings, *Miro1* knockout impaired mitochondrial transport into presynaptic regions of parvalbumin interneurons or motor neurons, resulting in rapidly progressing neurological diseases and death within weeks [29, 42]. Moreover, mutations in Miro1 significantly suppressed neuroblast formation, leading to neurological symptoms [13, 42]. In addition to its role in transport, Miro1 modulates Drp1 activity, whereas changes in Drp1 expression do not affect Miro1 levels. This suggests that Miro1 acts as an upstream regulator of both mitochondrial trafficking and morphology, a function that has only recently been recognized. Although previous studies have shown that nutrient or oxidative stress can downregulate Miro1, the effects of prenatal glucocorticoid exposure on Miro1 were not fully understood. We identified five putative GR responsive element (GRE) in the promoter region of the *RHOT1* gene (which encodes Miro1), suggesting that ligand-bound GR suppress Miro1 expression (Supplementary Fig. 8). Further studies are needed to understand how glucocorticoids impair mitochondrial dynamics as a feature of prenatal stress-induced neuronal disorders.

Miro comprise two GTPase (the nGTPase and cGTPase) which surround a pair of Ca^2+^-binding EF-hand domains. The nGTPase domain of Miro is functionally and structurally distinct from the cGTPase and is critically required for viability, mitochondrial size, and distribution of mitochondria out of the neuronal soma regardless of the employed motor [43]. Our research reveals a novel function of the N-terminal GTPase domain of Miro1 in regulating mitochondrial Ca^2+^ homeostasis through ERMC formation, providing a new perspective on Miro1’s role in differentiating neurons. The GTPase domain utilizes GTP energy to facilitate Miro1 function, while the EF hand primarily regulates mitochondrial transport and velocity by binding and unbinding to Ca^2+^ [10]. Miro1 detaches from microtubules upon binding to Ca^2+^, abundant in synapses, to relocate mitochondria to areas of high energy demand [26]. While Miro1’s activity is highly dependent on Ca^2+^ concentration, the specific domain responsible for this regulation has been debated. Although some researchers have suggested that the EF hand domain of Miro1 is responsible for Ca^2+^-induced detachment and rapid MiST, others argue that Miro1 is not essential for Ca^2+^-induced mitochondrial transport [42, 44]. Interestingly, Nemani et al. report that elevated Ca^2+^induces MiST in HeLa and MEF cells, dependent on Miro1, but not Drp1 [26]. In addition, in primary neurons, Miro1 serves as a Ca^2+^-sensitive bifunctional regulator for both the motility and fusion-fission dynamics of the mitochondria [45]. Several studies, including ours, indicate that mitochondrial Ca^2+^ levels, rather than cytosolic Ca^2+^, have a more pronounced effect on mitochondrial fusion and fission. These discrepancies may be due to variations in experimental methodologies, conditions, and cell types. In neurons, which have high energy demands and extensive networks, the function of Miro1 is particularly critical. Overexpression of Miro1 enhances neuronal activity, while its downregulation leads to axonal transport defects and increased risk for AD, such as tau hyperphosphorylation [46]. In contrast, Miro1 upregulation resulted in kyphosis in muscle cells [43]. Furthermore, no changes were detected in mitochondrial respiratory function or membrane potential levels in Miro1 KO embryonic fibroblasts [47]. Despite these outcomes, neurons exhibited significant alterations with Miro1 expression change, suggesting Miro1’s role in regulating intracellular Ca^2+^ distribution is crucial in neuronal contexts. Our findings also indicate that the N-terminal GTPase domain of Miro1 is crucial for regulating mitochondrial velocity, attributed to its role in facilitating mitochondrial Ca^2+^ influx (Supplementary Fig. 9). Our results highlight the importance of Ca^2+^ homeostasis maintained by Miro1 and the ERMCs to mitochondrial dynamics, affecting transport efficiency and neuronal integrity in differentiating neuron under prenatal stress.

Many studies have demonstrated that glucocorticoids exchange the intracellular Ca^2+^ concentration under both physiological and pathological conditions. Despite the apparent interest in the relationship between mitochondrial functions and Ca^2+^ concentration under prenatal stress, the role of glucocorticoid in this process remains poorly understood. Thus, our study sheds light on the interplay between mitochondrial morphology and ERMC under prenatal stress. N-terminal GTPase domain of Miro1 upregulated mitochondrial Ca^2+^ levels by enhancing ERMC formation. Mutations in this domain are closely associated with ERMC formation and mitochondrial dysfunction in stem cells [14, 44]. Phosphorylation of the N-terminal GTPase domain by Polo kinase recruits Miro1 to ERMC [14]. In addition, the domain is critical for Miro1’s localization, binding to the MCU protein, and promoting ER-mitochondrial interactions. This enhanced ERMC formation regulates mitochondrial Ca^2+^ influx, underscoring the importance of N-terminal GTPase in Miro1 localization in ERMC [47], consistent with our results. Conversely, the EF-hand domain does not appear to play a significant role in this process, according to current knowledge. Despite conflicting reports, our previous work and Park et al. constantly demonstrated that glucocorticoid exposure upregulated mitochondrial Ca^2+^ levels by increasing ERMC formation [48, 49]. Excessive ERMC formation is common in neurodegenerative disorders such as AD. In Parkinson’s disease, reduced Miro1 levels are associated with decreased ERMCs. Miro1 restoration can normalize trafficking in affected neurons but does not fully explain changes in mitochondrial morphology [47, 50, 51]. In addition, Miro interacts with Ca^2+^ transporters at the ERMCs. Its inactivation causes mitochondrial Ca^2+^ depletion and metabolic impairment, whereas its overexpression results in mitochondrial Ca^2+^ overload, mitochondrial morphology change, and apoptotic response [14, 52]. Based upon these results, we assumed that both prenatal and chronic stress lead to Miro1 reduction, but ERMC states differ, indicating that Miro1’s role in mitochondrial dynamics may vary depending on neuron maturity. Our findings demonstrated that Miro1 acts as a master regulator of mitochondrial fusion/fission by modulating ERMC formation. In terminally differentiated neurons, Miro1 may depend on other upstream molecules for its function. Future research should d explore how the N-terminal GTPase of Miro1 induces ERMC-mediated mitochondrial Ca^2+^ influx in differentiating neurons.

Our results demonstrate that reduced ERMC due to Miro1 downregulation leads to increased Drp1 activity, highlighting a novel mechanism by which prenatal stress induces abnormal mitochondrial fission. Indeed, both mouse hippocampal neurons and human iPSC-derived neurons exposed to prenatal glucocorticoid exhibited Drp1-mediated excessive mitochondrial fission. Mitochondrial fission is generally advantageous for differentiating neurons but can be detrimental when excessive, reducing dendritic mitochondrial content and [53], as observed in many neurodegenerative diseases such as AD [54]. Our results showed that manipulation of intracellular Ca^2+^ levels led to mitochondrial morphology alterations mediated by changes in Drp1 activity. According to previous reports, intracellular Ca^2+^ levels can affect Drp1 phosphorylation (Ser616) because Ca^2+^-dependent kinases such as MAPK or CaMK can activate Drp1. Mitochondrial division also usually occurs around ERMC sites by Miro1 or Mfn proteins, followed by recruitment of Drp1 [3, 55]. Furthermore, Miro1/2-dependent formation of mitochondrial-derived vesicles can be promoted by stimulating the subsequent recruitment of Drp1 in neurons, suggesting that Miro1 is an upstream signaling regulator for Drp1 [56]. There is another evidence that Miro1 can directly or indirectly influence Drp1 activity; Miro1 can recruit Drp1 to the mitochondrial surface to facilitate the fission to meet energy demands [57]. Miro1 can also promote or inhibit Drp1 activity depending on the cellular context and specific signaling pathways involved [45]. However, mitochondrial Ca^2+^ did not induce any morphological changes in dopaminergic neurons [58]. Similarly, alteration of Miro1 did not change mitochondrial morphology but evoked mitophagy in mature neurons [14]. Investigating the interaction between Miro1 and Drp1 involved in mitochondrial dynamics will enhance our understanding of the regulatory mechanisms depending on neuron type or maturity because their relationship is multifaceted. Thus, further research is necessary to understand why the types of neurons and the stages of differentiation can change the upstream molecules for regulating mitochondrial dynamics. Behavioral analyses revealed that Miro1 restoration in mice exposed to prenatal stress normalized mitochondrial distribution and synaptic function. It resulted in recovered cognitive and reduced depressive/anxiety-related behaviors, while no improvement was seen with the overexpression of the N-terminal GTPase mutant. These findings underscore the potential of targeting Miro1 to mitigate the long-term effects of prenatal stress on brain function. Our study highlights the critical role of Miro1, particularly its N-terminal GTPase domain, in regulating mitochondrial dynamics in differentiating neurons exposed to prenatal glucocorticoids. By decreasing ERMC formation and modulating Drp1 activity, Miro1 ensures proper mitochondrial fusion/fission balance, essential for neuronal development and function. These findings provide a new perspective on the significance of Miro1 in prenatal stress contexts and its potential as a therapeutic target for preventing neurodevelopmental and neuropsychiatric disorders.

To our knowledge, this is the first study to establish a direct link between Miro1 and stress-related mitochondrial dynamics. While previous research has demonstrated that chronic stress can induce mitochondrial fission through Drp1 activation, no prior studies have implicated Miro1 in mitochondrial dysfunction induced by both prenatal and adult stress. Thus, our findings uniquely highlight Miro1 as a key mediator of mitochondrial fusion/fission imbalance under prenatal stress conditions, the expression of which was not changed in differentiated neurons, providing new insights into the molecular mechanisms underlying stress-induced neurodevelopmental disorders. Despite these novel discoveries, several limitations must be acknowledged, particularly in terms of clinical translation. One major challenge is the feasibility of early interventions. Implementing gene therapy or pharmacological treatments for manipulating Miro1 upregulation immediately after birth may be ethically complex. Additionally, the long-term effects and safety of targeting mitochondrial dynamics in developing neurons remain unclear. Future research should focus on determining whether postnatal therapeutic applications exist to effectively mitigate mitochondrial dysfunction caused by prenatal stress, reducing the risk of neurodevelopmental disorders later in life.

Our findings suggest that Miro1-mediated suppression of Drp1 impacts mitochondrial dynamics, potentially leading to impaired respiratory function and disrupted mitochondrial quality control. Mitochondrial fission could induce reduced oxygen rate consumption, glucose tolerance and thermogenesis where such dysregulation has been implicated in various diseases including metabolic syndrome, aging-related senescence, cancer, and neurodevelopmental/neurodegenerative disorders [59–61]. These detrimental effects induced by dysfunctional mitochondrial dynamics can exaggerate prenatal stress-induced neurodegeneration. Prenatal stress-induced alterations in mitochondrial morphology may contribute to long-term developmental and metabolic abnormalities, as mitochondria play a critical role in cellular energy homeostasis and glycolysis/OXPHOS regulation.

## Conclusion

Our study shows that restoration of Miro1, particularly the N-terminal GTPase domain responsible for the formation of ERMCs, is a critical mechanism to protect neuronal development from dysfunction of mitochondrial dynamic through Drp1 phosphorylation (Ser616) in differentiating neurons exposed to prenatal glucocorticoids.

## Electronic supplementary material

Below is the link to the electronic supplementary material.

Supplementary Material 1

Supplementary Material 2

Supplementary Material 3

## Data Availability

No datasets were generated or analyzed during the current study.

## Abbreviations

AD: Alzheimer’s Disease
Drp1: Dynamin-related Protein 1
ER: Endoplasmic Reticulum
ERMC: ER-mitochondria Contact
FIS1: Mitochondrial Fission 1 Protein
GR: Glucocorticoid Receptor
hiPSC: Human Induced-Pluripotent Stem Cell
IP3R: Inositol 1,4,5-Trisphosphate Receptor
MAM: Mitochondria-Associated Membrane
MCU: Mitochondrial Calcium Uniporter
Miro1: Mitochondrial Rho GTPase 1
Miro2: Mitochondrial Rho GTPase 2
MiST: Mitochondrial Shape Transition
Mfn: Mitofusins
MTG: MitoTracker Green
MTR: MitoTracker Red
NSCs: Neural Stem Cells
NOR: Novel Object Recognition
Opa1: Optic Atrophy 1
TOMM20: Translocase of Outer Mitochondrial Membrane 20
TMRE: TetraMethylRhodamine Ethyl ester
VDAC1: Voltage-Dependent Anion Channel 1
