# Supplementary figures and images for: Restoration of Miro1’s N-terminal GTPase function alleviates prenatal stress-induced mitochondrial fission via Drp1 modulation
Source: Cell Commun Signal. 2025 Apr 2;23:166. doi: 10.1186/s12964-025-02172-5 (PMC11967123; doi:10.1186/s12964-025-02172-5)

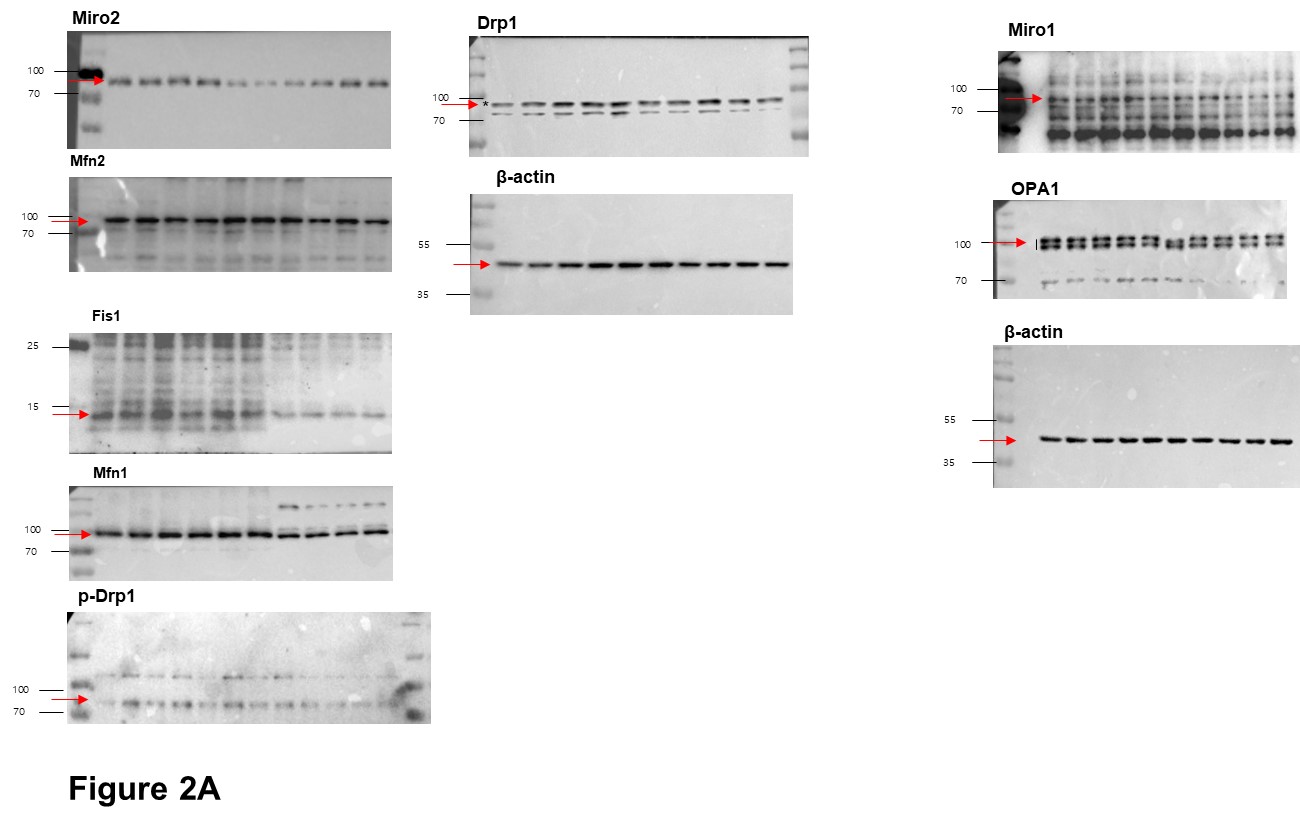


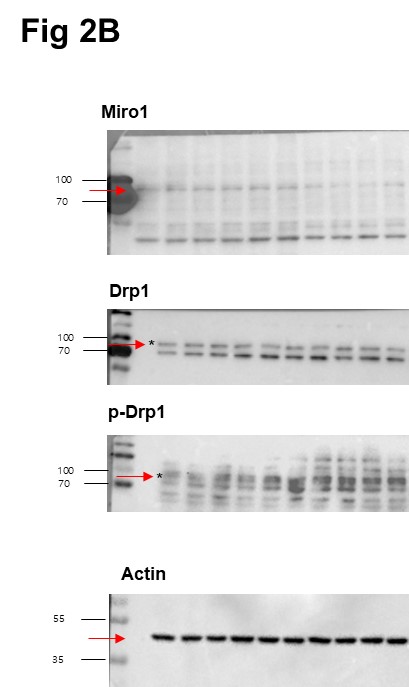


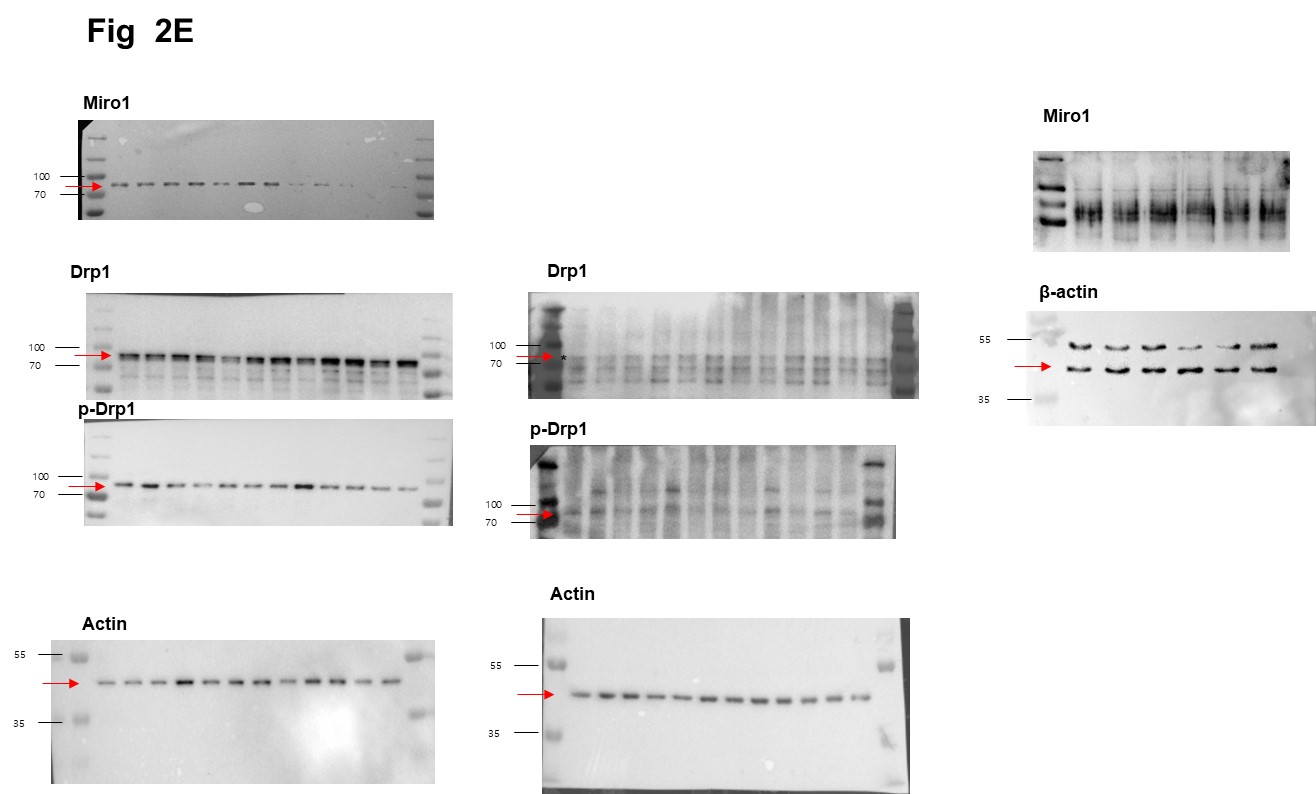


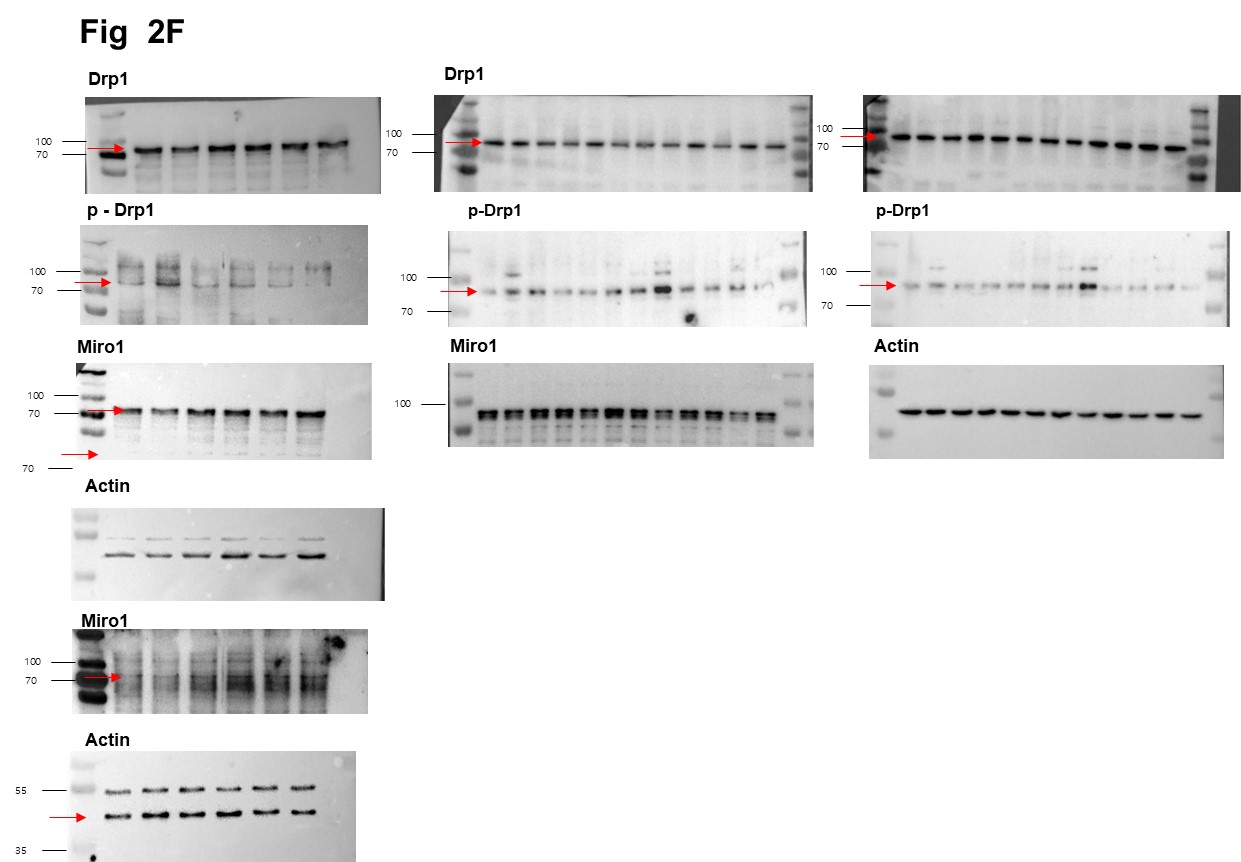


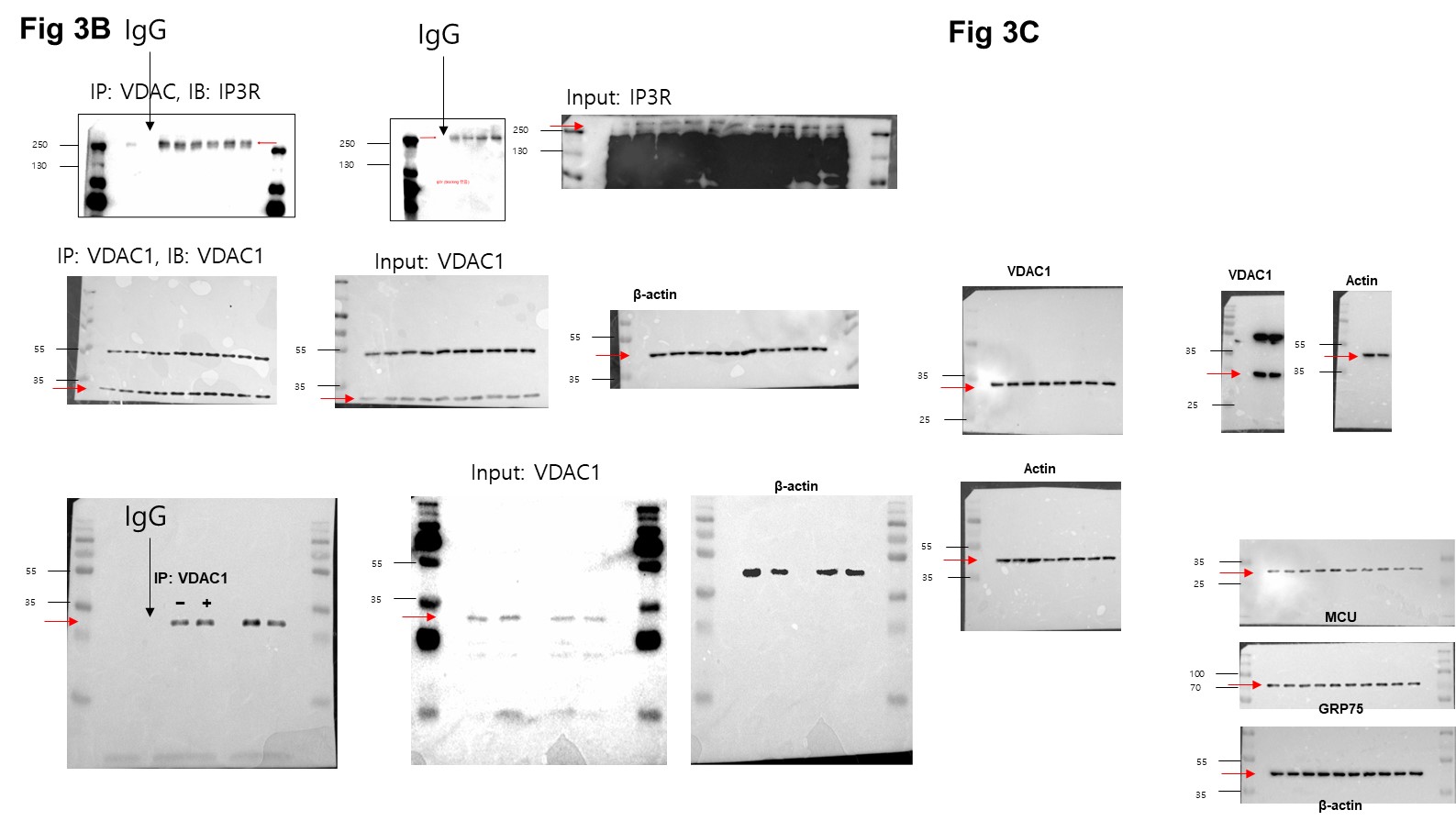


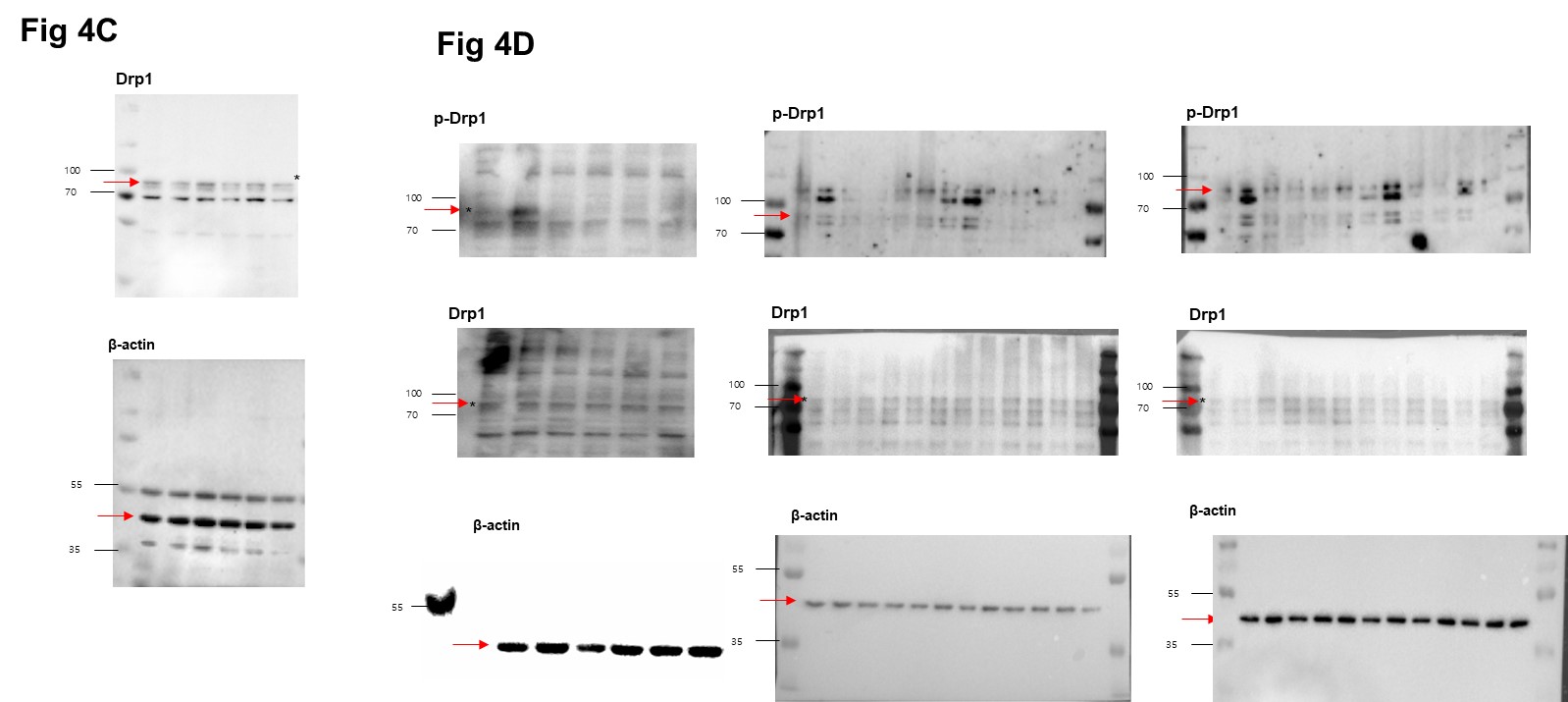


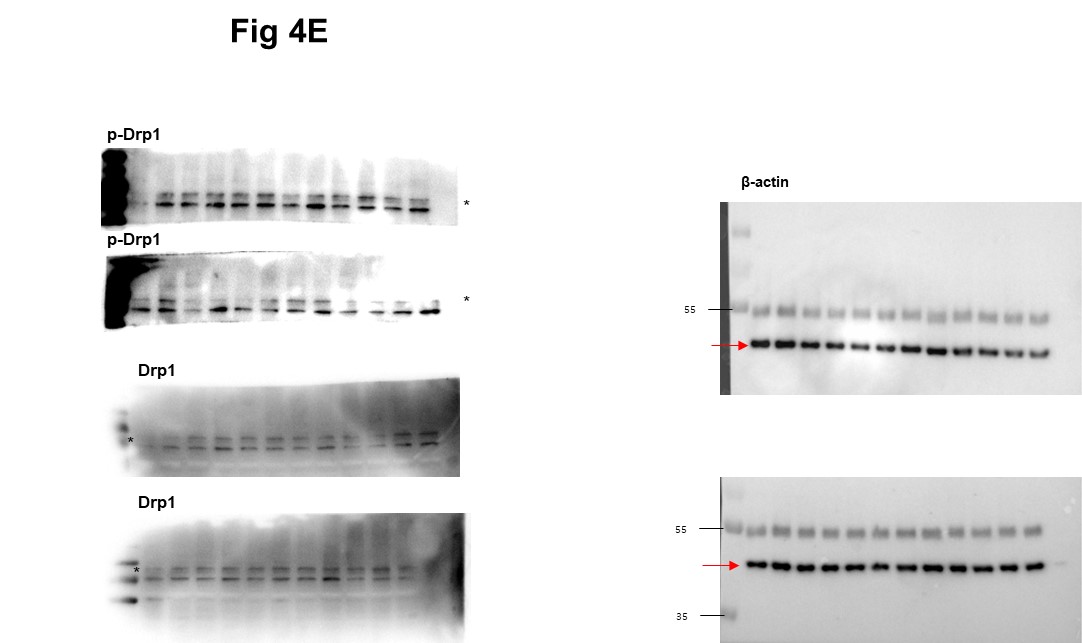


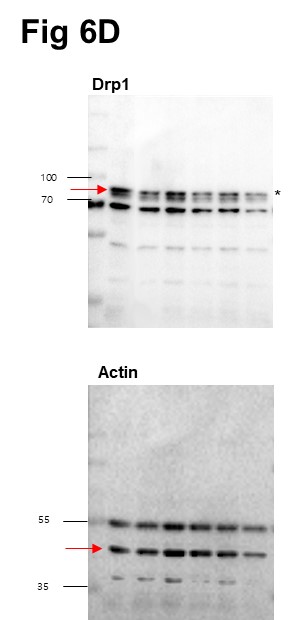


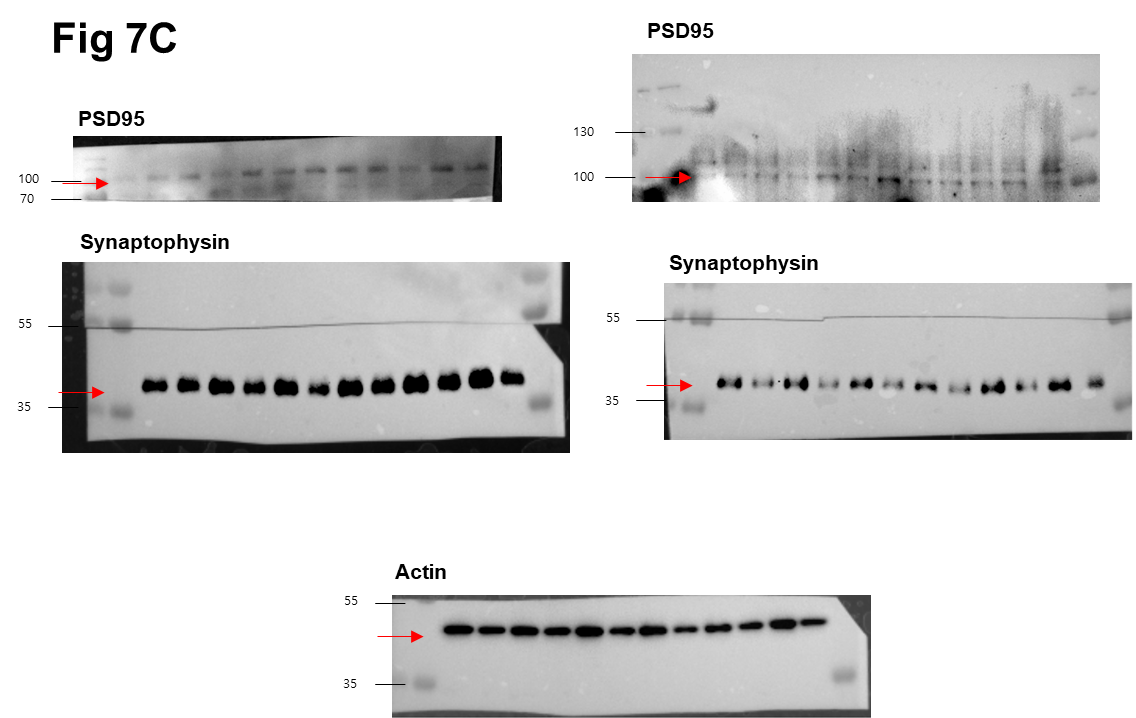


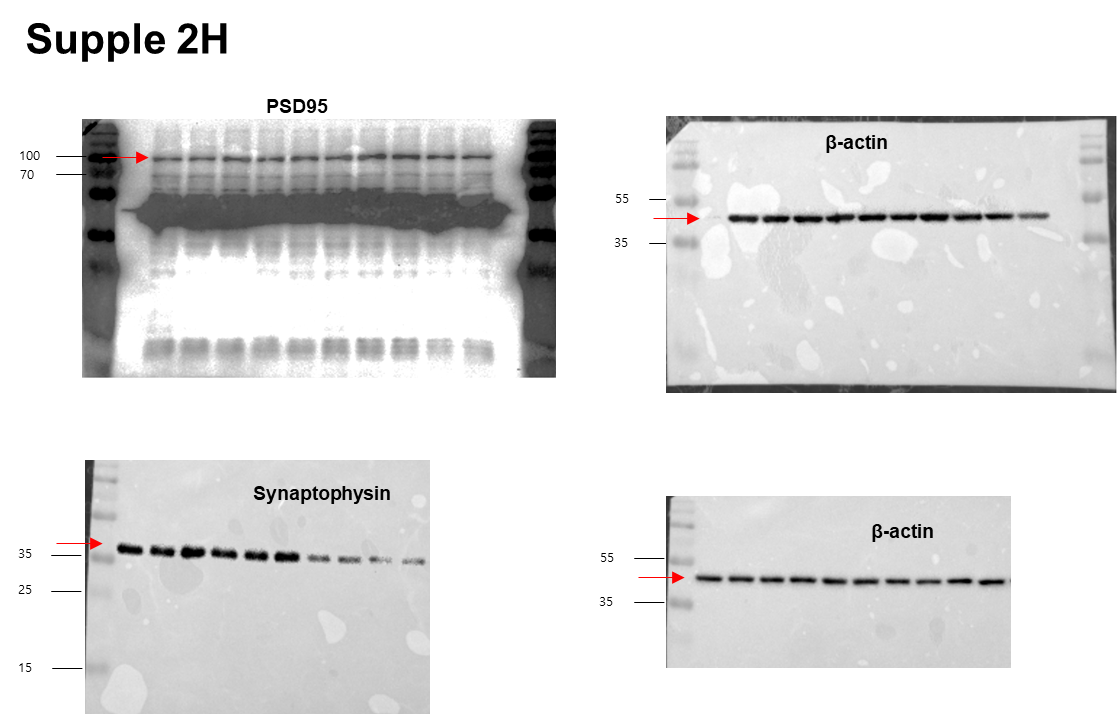


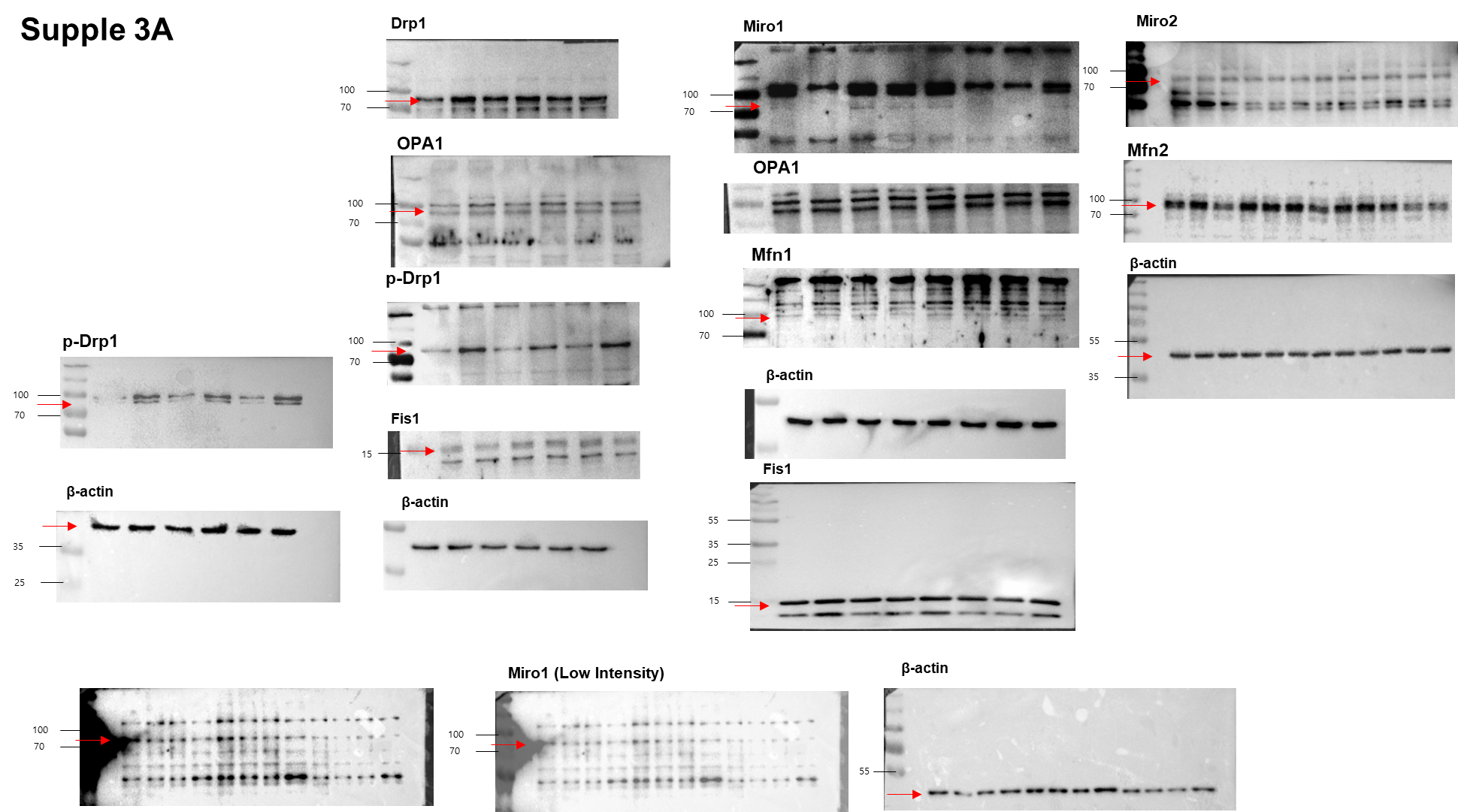


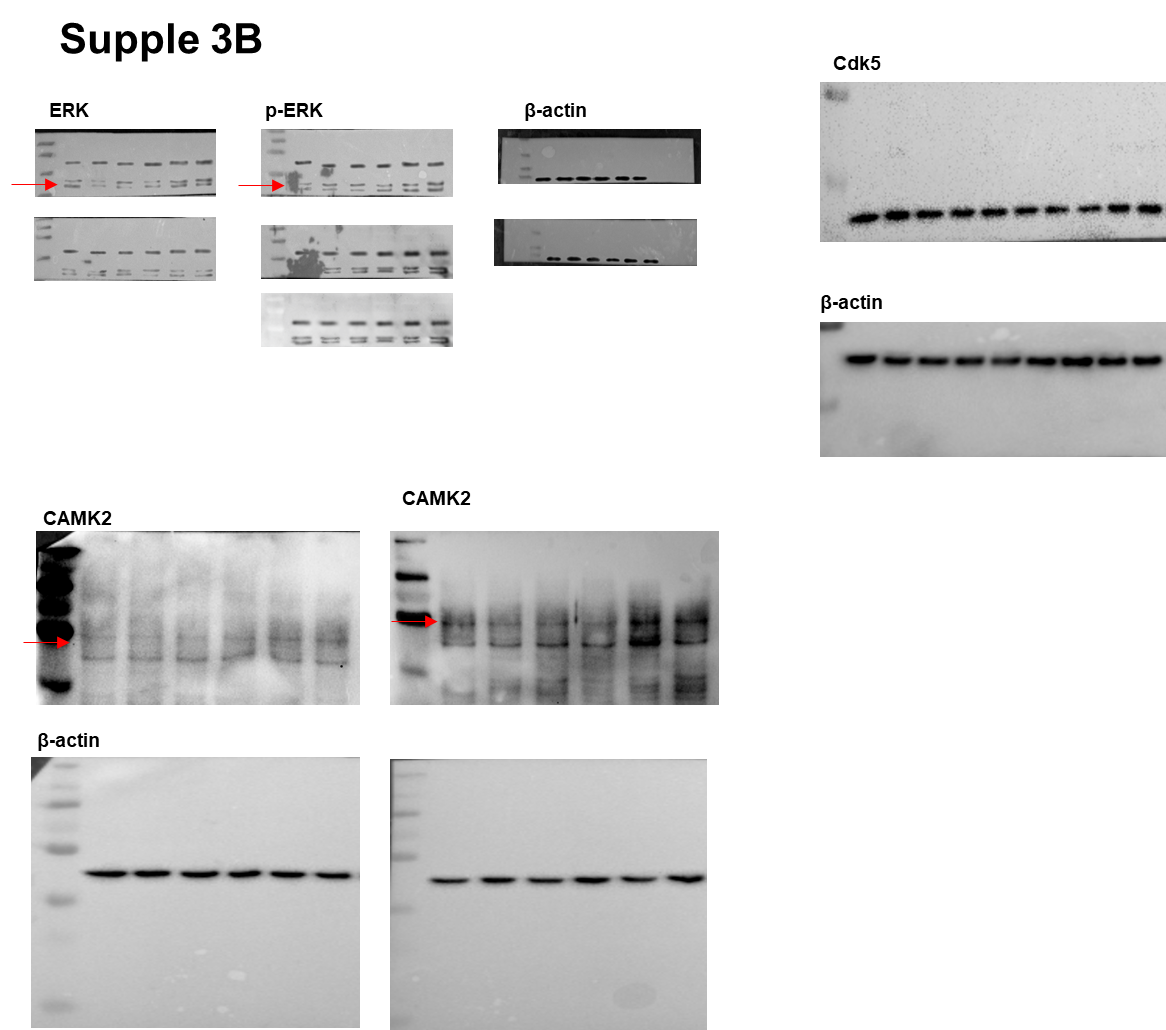


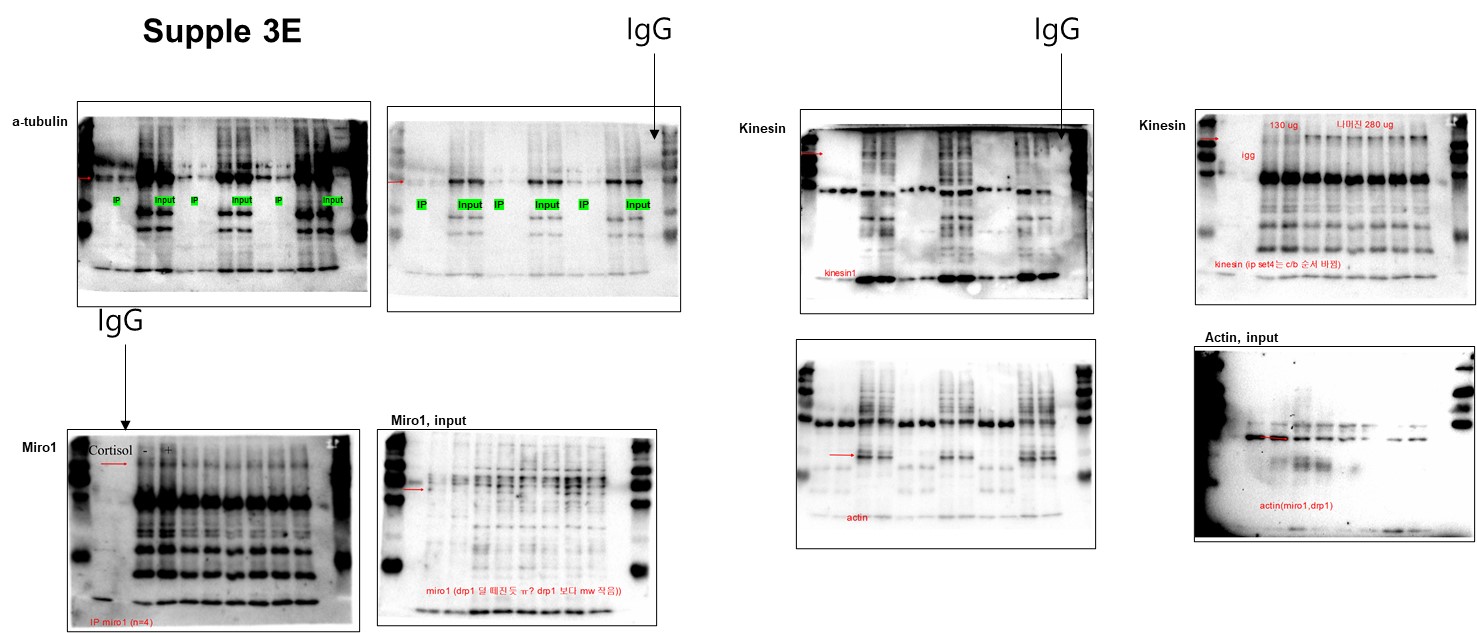


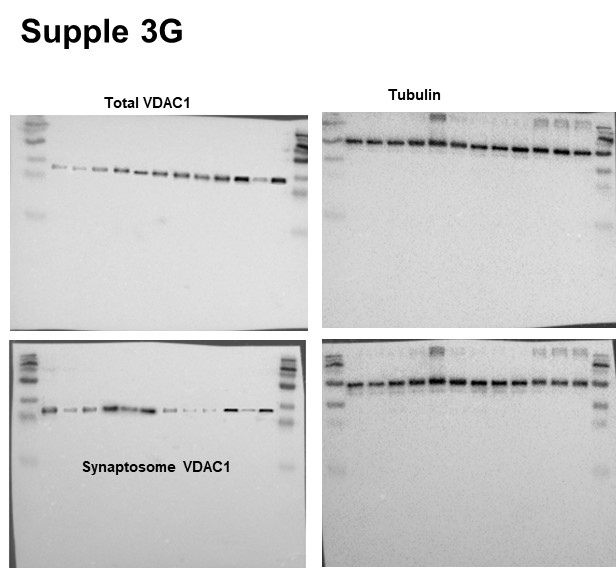

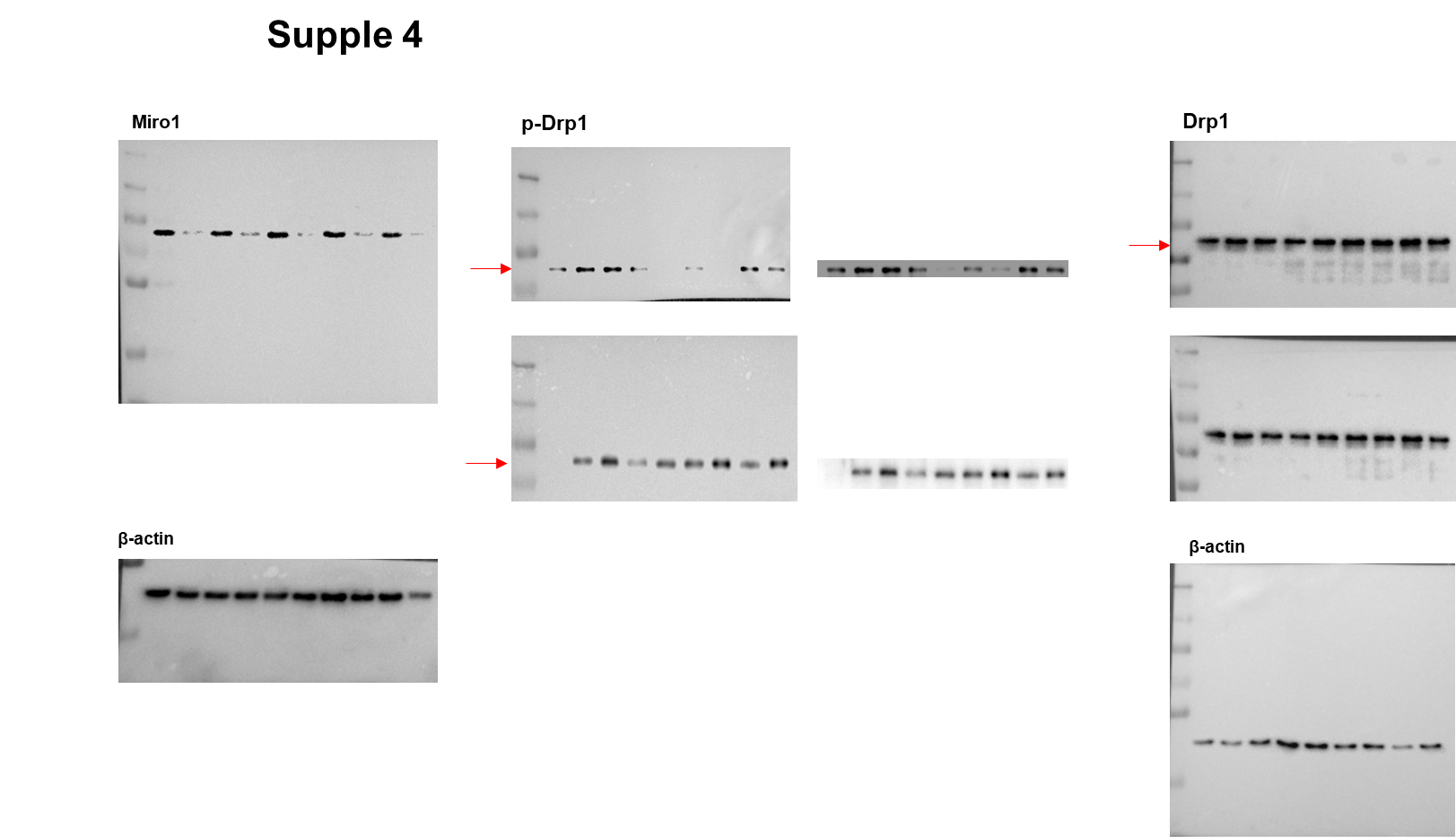

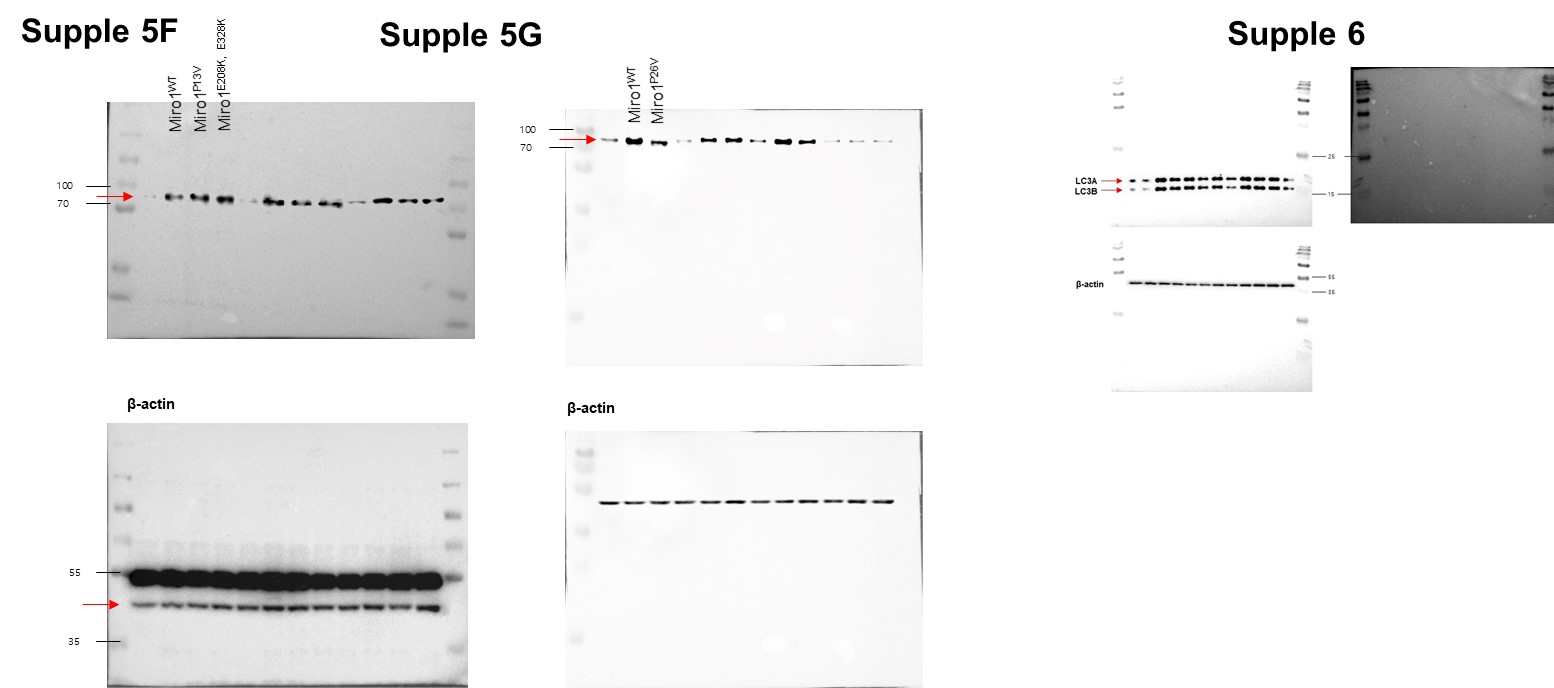

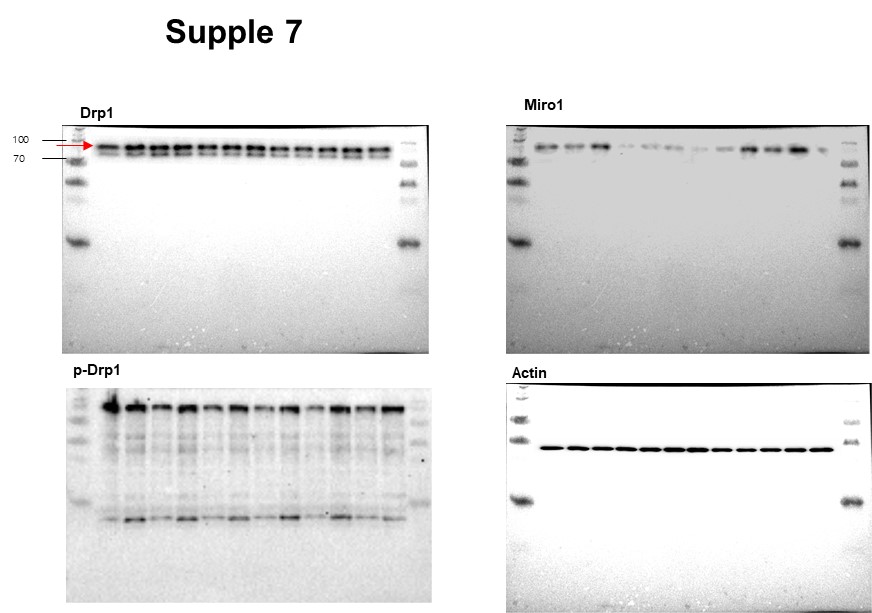

Supplement: Supplementary file 1 — Supplementary Material 1 [file 12964_2025_2172_MOESM1_ESM.docx]
