# Supplementary material for: Restoration of Miro1’s N-terminal GTPase function alleviates prenatal stress-induced mitochondrial fission via Drp1 modulation
Source: Cell Commun Signal. 2025 Apr 2;23:166. doi: 10.1186/s12964-025-02172-5 (PMC11967123; doi:10.1186/s12964-025-02172-5)
Supplement: Supplementary file 2 — Supplementary Material 2 [file 12964_2025_2172_MOESM2_ESM.docx]

**SUPPLEMENTARY information FOR**

**Restoration of Miro1’s N-terminal GTPase function alleviates prenatal stress-induced mitochondrial fission via Drp1 modulation**

Gee Euhn Choi^2, 3, #^, Ji Yong Park^1, #^, Mo Ran Park^1, #^, Chang Woo Chae^4^, Young Hyun Jung^5^, Jae Ryong Lim^1^, Jee Hyeon Yoon^1^, Ji Hyeon Cho^1^, and Ho Jae Han,^1, *^

^1^ Department of Veterinary Physiology, College of Veterinary Medicine, Research Institute for Veterinary Science, BK21 FOUR Future Veterinary Medicine Leading Education & Research Center, Seoul National University, Seoul, 08826, South Korea.

^2^ Laboratory of Veterinary Biochemistry, College of Veterinary Medicine and Veterinary Medical Research Institute, Jeju National University, Jeju, 63243, South Korea.

^3^ Interdisciplinary Graduate Program in Advanced Convergence Technology & Science, Jeju National University, Jeju, 63243, South Korea

^4^ Department of Physiology and Medical Science, College of Medicine and Brain Research Institute, Chungnam National University, Daejeon 35015, South Korea

^5^ Department of Physiology, College of Medicine, Soonchunhyang University, Cheonan, 31151, Republic of Korea

^#^ These authors contributed equally

^*^ Correspondence: hjhan@snu.ac.kr


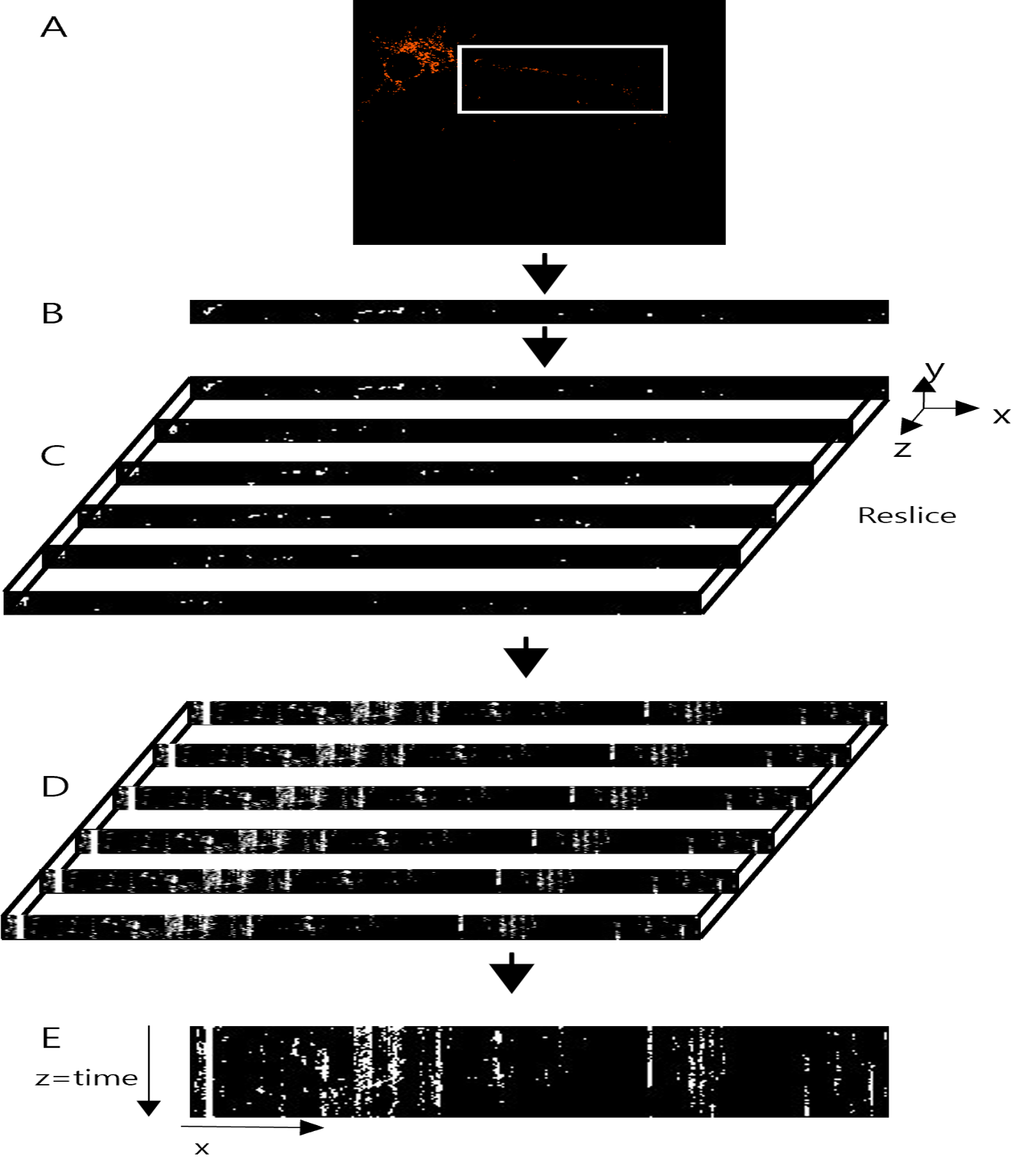


**Supple Fig. S1. Schematic of kymograph creation**

Human NSCs and mouse hippocampal neurons was immunostained with MTR (red) and time-lapse imaging of mitochondrial movement were recorded for 5 minutes. (A-B) The axon of neuron was selected and straightened using the straighten plug-in by using Image J. (C-D) Reulting image stacks were subsequently resliced along the plane, resulting in a x-z view of the time series (5 min). (E) The kymograph was then created by a z-projection of resliced time series using z-projection plug-in.


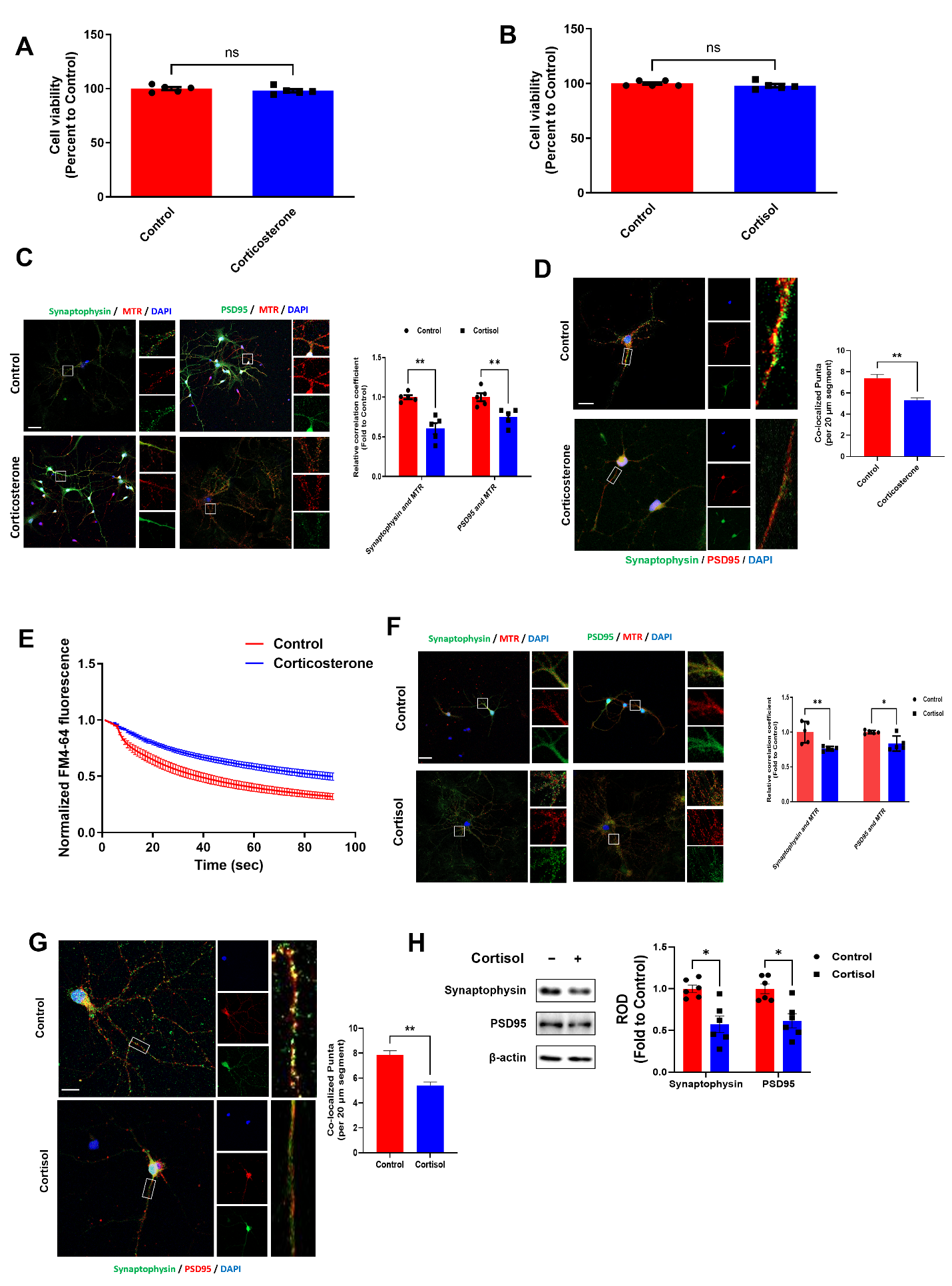


**Supple Fig. S2. Prenatal glucocorticoid exposure adversely affects synaptic function**

(A,C – F). Female pregnant mice were exposed to either vehicle or corticosterone (10 mg/kg) at E14 and mouse hippocampal neurons from E18 fetus were cultured until DIV14. (A) WST-1 assay was performed to measure cell viability. N=5. (B,G – H) Human NSCs were cultured in neuronal differentiation media for 6 days and treated with cortisol (1 μM) for 48 h. (B) WST-1 assay was performed to measure cell viability. N=5. (C) Hippocampal neurons were immunostained with PSD95 (green), synaptophysin (blue), and MTR (red). Pearson’s correlation coefficient was quantified for mitochondrial distribution in synapse. Scale bars, 20 μm (magnification, x 1,000). n=5. (D) Quantification of the number of synaptophysin/PSD95 puncta pairs per 20 µm. Scale bars, 20 μm. The number of neurites extending from the soma of 20 neurons. *n*=5. (E) Hippocampal neurons were stained with FM4-64 dye and stimulated with high K^+^ buffer for destaining. Time-lapse imaging was done over 90 sec at 1 sec intervals with an Eclipse Ts2TM fluorescence microscopy. n=5. (F) Human NSCs were immunostained with PSD95 (green/red), Synaptophysin (green), MTR (red), and DAPI (blue). Pearson’s correlation coefficient was quantified for detecting pre/post-synaptic mitochondria distribution. Scale bars, 20 μm. n=5. (G) Quantification of the number of synaptophysin/PSD95 puncta pairs per 20 µm. Scale bars, 10 μm. The number of neurites extending from the soma of 20 neurons. (H) The expressions of PSD95 and synaptophysin were determined by western blot. The β-actin was used as a loading control. n=5.


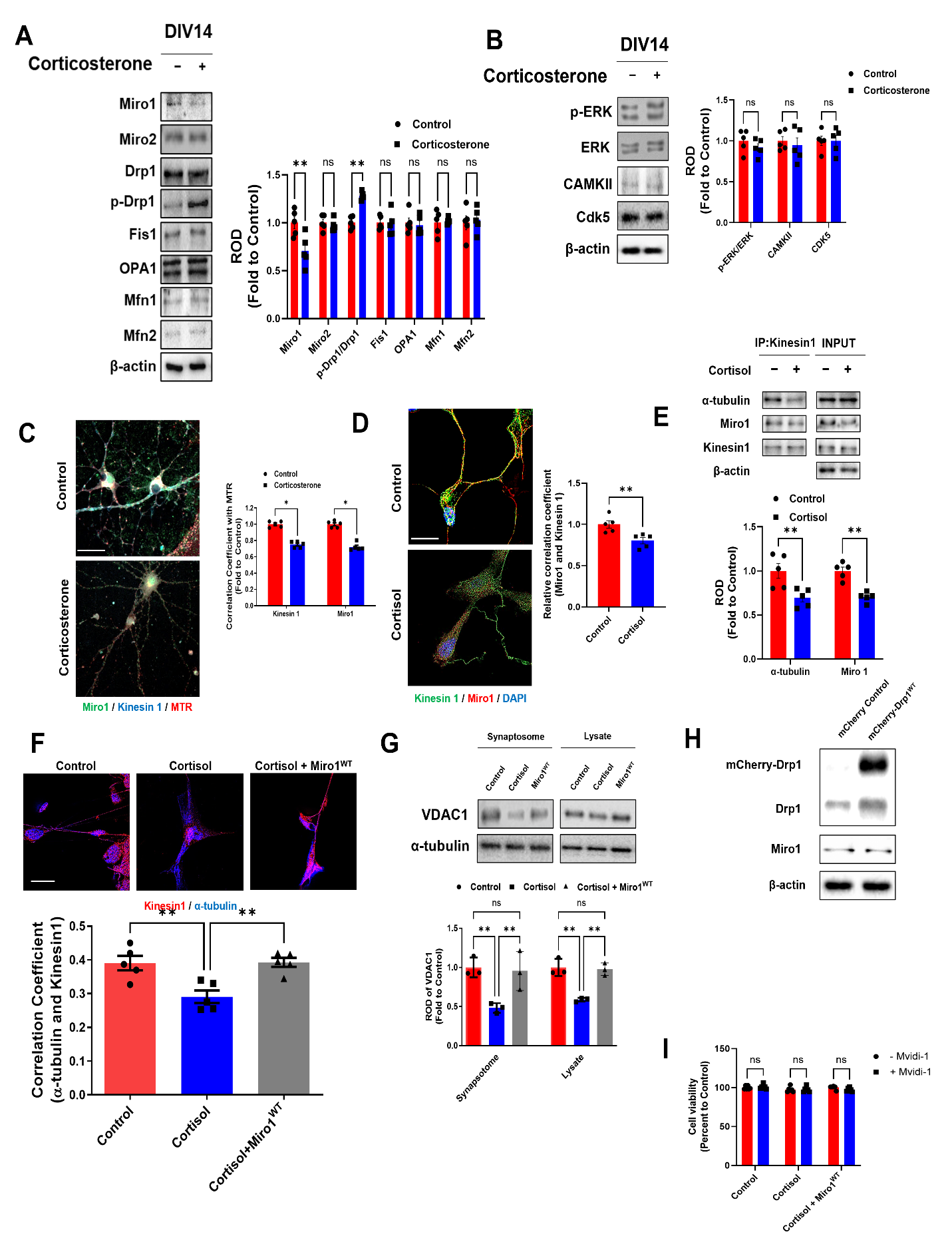
 **Supple Fig. S3. Miro1 downregulation triggered defects in mitochondrial trafficking and motility.**

(A) Female pregnant mice were exposed to either vehicle or corticosterone (10 mg/kg) at E14 and mouse hippocampal neurons from E18 fetus were cultured until DIV14. The expressions of Miro1/2, Drp1, p-Drp1 (S616), Fis1, OPA1, and Mfn1/2 were determined by western blot. The β-actin was used as a loading control. *N*=5. (B) The expression of ERK, p-ERK, CAMKII, and CDK5 were determined by western blot. The β-actin was used as a loading control. *N*=5. (C) Hippocampal neurons were immunostained with Miro1 (green), kinesin 1 (blue), and MTR (red). Pearson’s correlation coefficient was quantified. Scale bars, 20 μm. n=5. (D – E) Human NSCs were cultured in neuronal differentiation media for 6 days and treated with cortisol (1 μM) for 48 h. (D) Human NSCs were immunostained with kinesin 1 (green), Miro1 (red), and DAPI (blue). Pearson’s correlation coefficient was quantified for detecting interaction between Miro1 and kinesin 1. Scale bars, 20 μm. n=5. (E) Kinesin 1 was co-immunoprecipitated with α-tubulin and Miro1. The α-tubulin and Miro1 levels in immunoprecipitated samples were quantified. n=5. (F) Human NSCs were transduced with Miro1WT expression vector 24 h after seeding. Human NSCs were immunostained with α-tubulin (blue) and kinesin 1 (red). Pearson’s correlation coefficient was quantified between α-tubulin and kinesin 1. Scale bars, 20 μ. n=5. (G) The expression of VDAC1 and α-tubulin in synaptosome were detected by western blot. (H) Western blot analysis confirms the overexpression of mCherry-Drp1 WT and mCherry control in hiPSC-derived neuron. The expression of Drp1 and Miro1 were determined by western blot. The β-actin was used as a loading control (I) WST-1 assay was performed to measure cell viability. n=5. Quantitative data are presented as a mean ± S.E.M. The representative images were acquired by SRRF imaging system. *, ** indicates p < 0.05, p < 0.01 versus control, respectively. #, ## indicates p <0.05, p <0.01 versus cortisol in human NSCs, respectively.


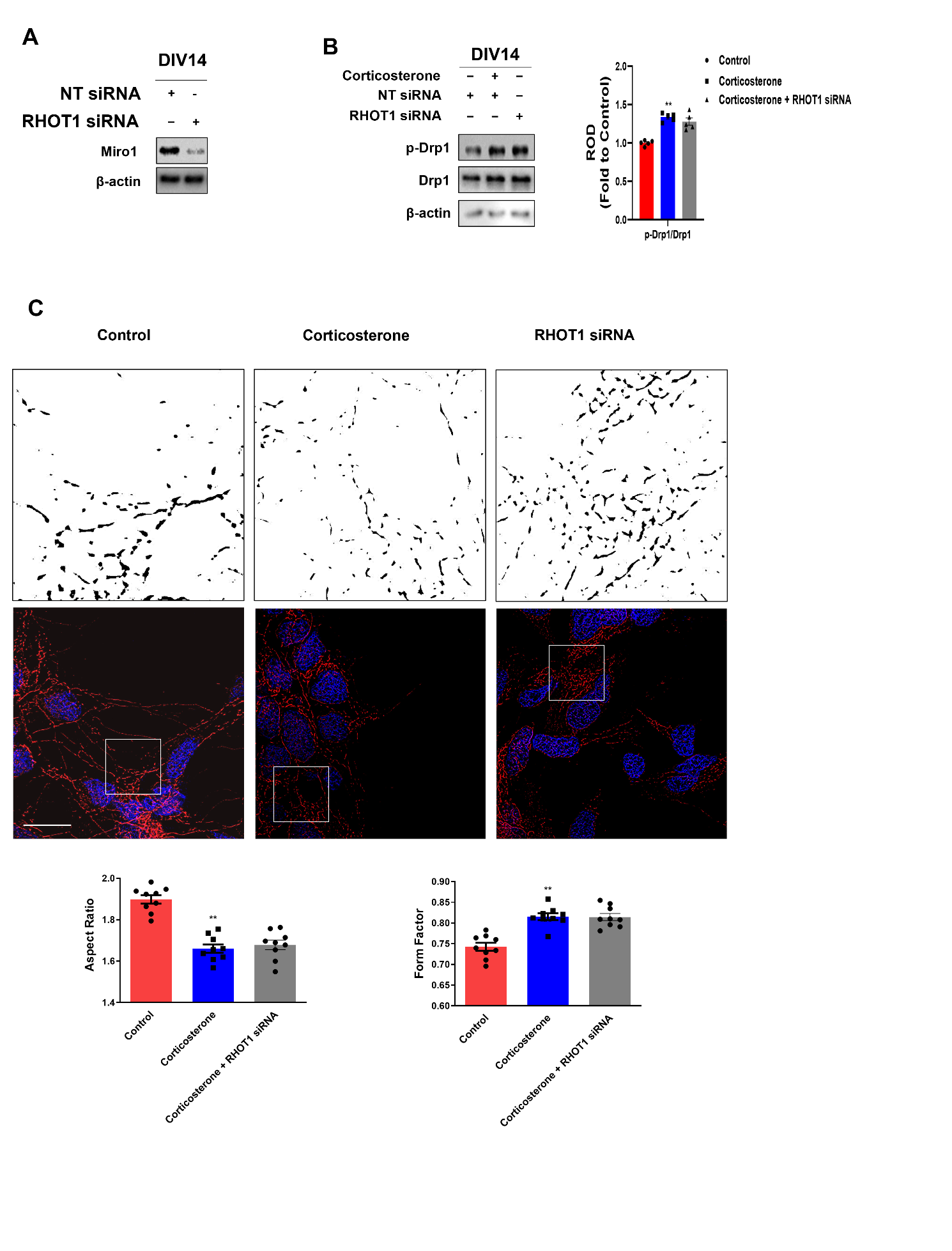


**Supple Fig. S4. The effect or Miro1 silencing in mitochondria arrest and excessive fission.**

Female pregnant mice were exposed to either vehicle or corticosterone (10 mg/kg) at E14 and mouse hippocampal neurons from E18 fetus. Nontargeting (NT) or *rhot1* siRNA was transfected 48 h before imaging and blotting. (A) The expressions of Miro1 were determined by western blot. The β-actin was used as a loading control. n=5. (B) The expression of p-Drp1 and Drp1 were determined by western blot. The β-actin was used as a loading control. n=5. (C) Quantification of mitochondrial shape descriptors (form factor and aspect ratio) were determined by using Fiji software. Scale bars, 20 μm (magnification, x 1,000). N=9. Quantitative data are presented as a mean ± S.E.M. The representative images were acquired by SRRF imaging system. ** indicates p < 0.01 versus control.


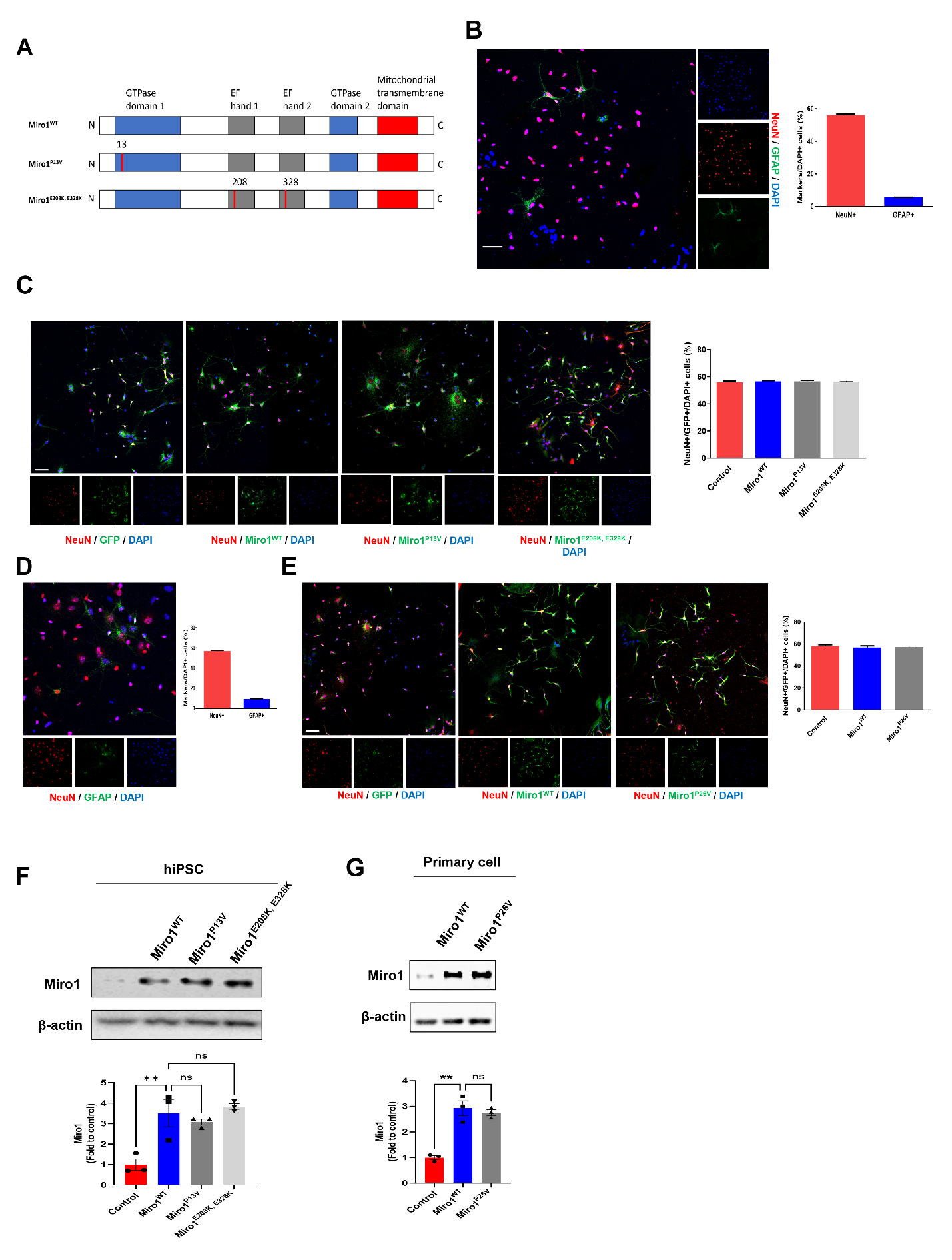


**Supplementary Fig S5. Efficiency of Miro1 varients transfection.** (A). Schematic of the primary structure of Miro1. (B) Purity of iPSC-derived neuron. Human NSCs were immunostained with NeuN (red), GFAP (green), and DAPI (blue) and quantified by calculating the ratio between NeuN+/GFAP+ cells and total number of cells. Scale bar 100 μm. n=30 (C). Efficiency of Miro1 varients in iPSC-derived neuron. Human NSCs were immunostained with NeuN (red), GFP (green), and DAPI (blue) and quantified by calculating the ratio between NeuN+/GFP+ cells and total number of cells. (D) Efficiency of Miro1 varients in primary hippocampal neuron. Primary hippocampal neurons were immunostained with NeuN (red), GFP (green), and DAPI (blue) and quantified by calculating the ratio between NeuN+/GFP+ cells and total number of cells. (E) Purity of primary hippocampal neuron. Primary hippocampal neurons were immunostained with NeuN (red), GFAP (green), and DAPI (blue) and quantified by calculating the ratio between NeuN+/GFAP+ cells and total number of cells. Scale bar 100 μm. n=30 (F) Human NSCs were transfected with Miro1^WT^, Miro1^P13V^, and Miro1^E208K, E328K^ expression vector 24 h after seeding. The expressions of Miro1 were determined by western blot. The β-actin was used as a loading control. (G) Mouse hippocampal neurons were transfected with Miro1^WT^ and Miro1^P26V^ expression vector 48 h before imaging and blotting. The expressions of Miro1 were determined by western blot. The β-actin was used as a loading control.


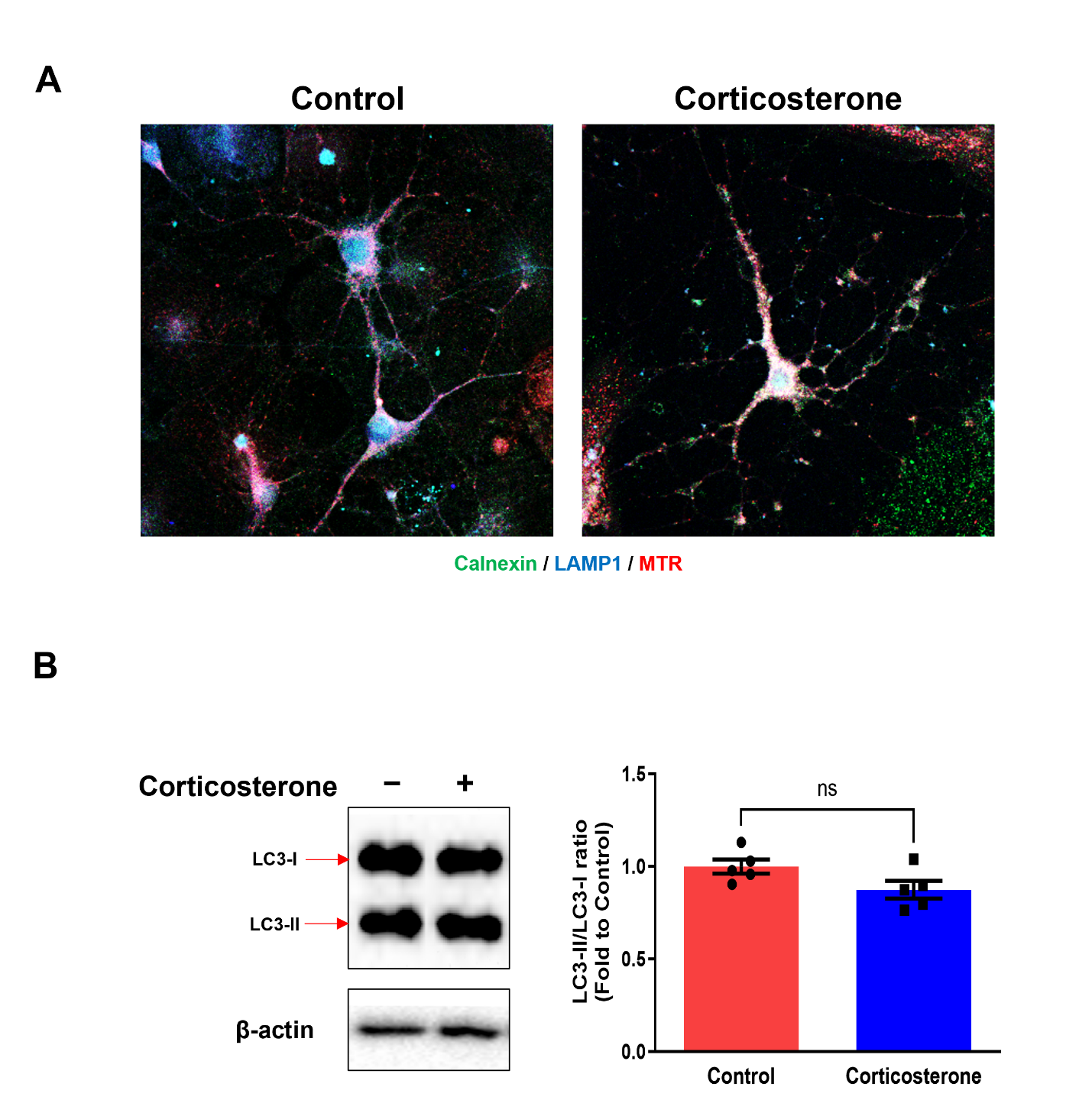


**Supplementary Fig S6. The effects of prenatal corticosterone on mitophagy and autophagy.** Female pregnant mice were exposed to either vehicle or corticosterone (10 mg/kg) at E14 and mouse hippocampal neurons from E18 fetus were cultured until DIV14. (A) Hippocampal neurons were stained with calnexin (green), LAMP1 (blue), and MTR (red). (B) The expressions of LC3 were determined by western blot. The β-actin was used as a loading control. n=5

**
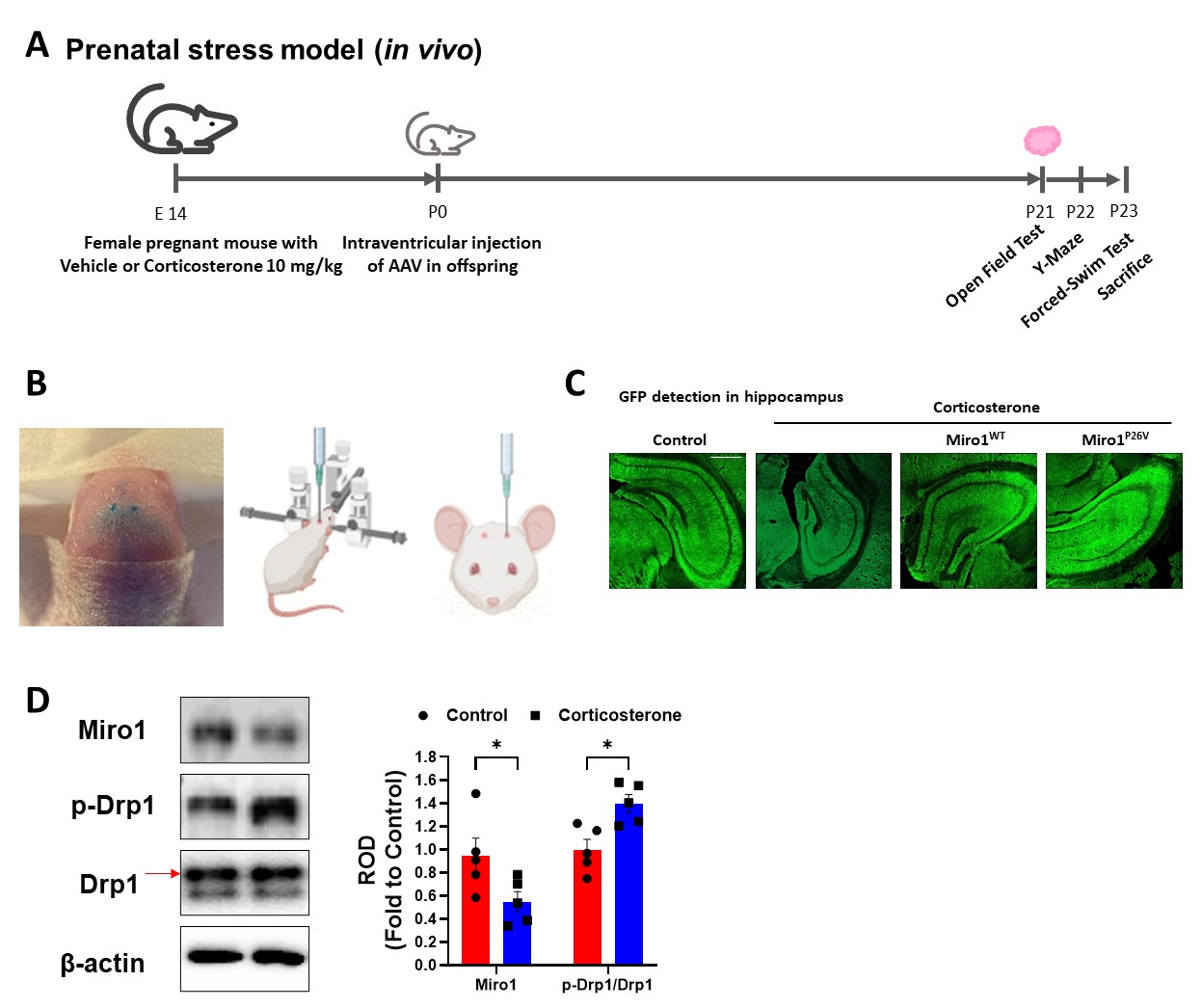
**

**Supple Fig. S7.** **Schematic of stereotaxic injection of P0 mice.** (**A**) The brief schedule of *in vivo* study was shown. (**B**) The head of P0 mice which received AAV injections at lateral ventricle was presented. (**C**) The representative images of GFP fluorescence detection in hippocampus were presented. Scale bars, 100 μm. (D) The expression of p-Drp1, Drp1 and Miro1 in brain of offspring were determined by western blot. The β-actin was used as a loading control. n=5


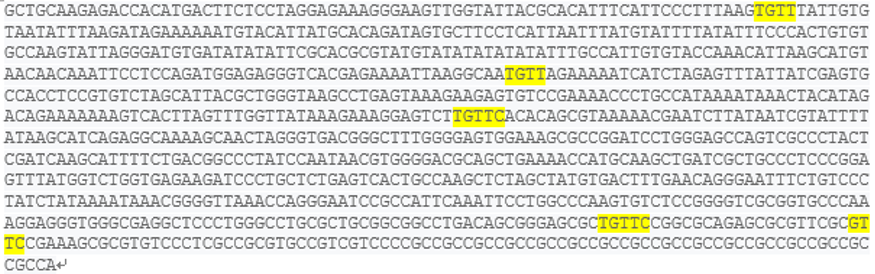


**Supple Fig. S8. The promoter regions of Miro1.** A thousand base pair upstream of the first codon of the *RHOT1* was described and the putative GRE binding sequence was emphasized with yellow labeling.


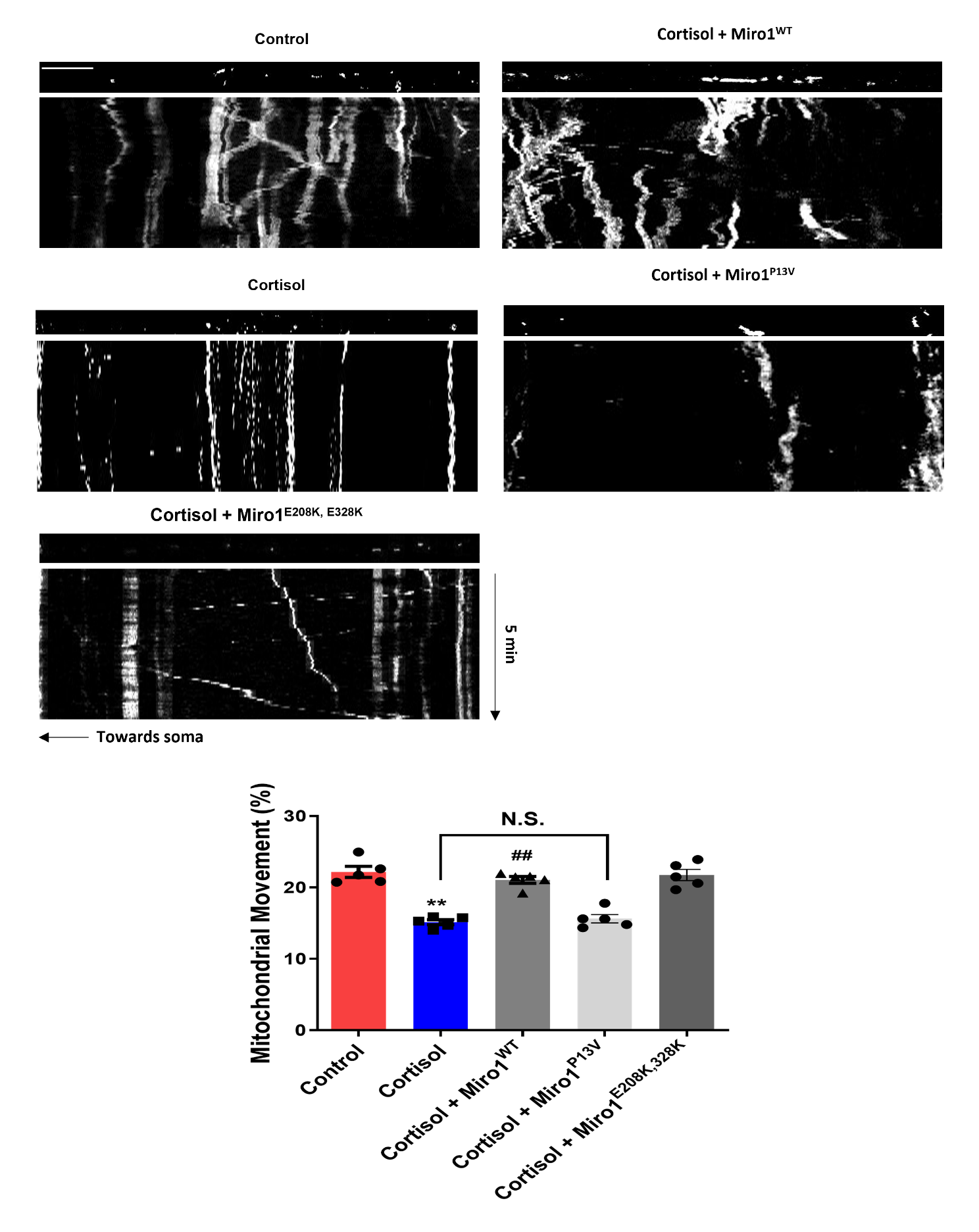
**Supple Fig. S9.** **Effects of prenatal glucocorticoid exposure and Miro1 mutation on mitochondrial dynamics in differentiating neurons.** Human NSCs were transduced with Miro1^WT^, Miro1^P13V^, and Miro1^E208K, E328K^ expression vector 24 h after seeding and stained with MTR (red) and time-lapse imaging of mitochondrial movement were recorded for 5 minutes. The first frame of the time-lapse image series is shown above of a kymograph generated form the movie. The x-axis corresponds to mitochondrial position and y-axis corresponds to time (progressing from top to bottom). Quantification of mitochondrial mobility in situ and velocity of moving mitochondria in the 5 min period using Fiji software. Scale bars, 10 μm. n=5
