# Supplementary material for: Restoration of Miro1’s N-terminal GTPase function alleviates prenatal stress-induced mitochondrial fission via Drp1 modulation
Source: Cell Commun Signal. 2025 Apr 2;23:166. doi: 10.1186/s12964-025-02172-5 (PMC11967123; doi:10.1186/s12964-025-02172-5)

Characterization of hPSC

**Cell Line Name**	**CMC-hiPSC-003**
**Type of Cell Line**	**hiPSC**
**Depositor (Institution)**	**Catholic University of Korea**
**Passage #**	**p20***
**Day of Cell Freezing**	**20221025***

Analysis	Result	Passage#	Day of analysis
Cell viability	Pass (91.3±1.5%)	p20	20221104
Attached cell number (after thawing 5 day)	1.35x10^7 cell/ml	p21	20221108
Authentication (STR)	Pass	p22	20221125
Mycoplasma test (PCR)	Pass	p22	20221108
Microbial contamination test (Virus, Fungi, bacteria)	Pass	p22	20221125
Karyotype (G-banding)	46,XY	p22	20221129
Cell attachment and colony morphology	Pass	p24	20200813
CNV analysis (CMA)	Not-detected	p22	20221122
ABO genotype	AO	p19	20221201
HLA genotype	HLA-A *33:03 *33:03<br>HLA-B *44:03 *44:03<br>HLA-DRB1*13:02 *13:02	p19	20221107
Stem Cell Marker Expression			
· AP staining	Pass (Positive)	p19	20221028
· ICC	Pass (Positive)	p19	20221024
· qRT-PCR	Pass (Positive)	p19	20221026
Differentiation Marker Expression			
· EB formation	Pass (EB14d)	p18	20221030
· qRT-PCR	Pass (Positive)	p18	20221031
· Teratoma formation	Pass	p18*(MCB)	20190505

* Freezing media : Stem Cell Banker

Cell Culture Condition

· Feeder/matrix	Vitronectin (Gibco, A14700)
· Media	TeSR-E8 (Stem Cell Technol, ST05940)
· Plask	T75
· Passage (Cell dissociation)	EDTA/Gentle Cell Dissociation Reagent (Stem cell Technol, 07174)

Description of the hPSC

· Parental Cell	Bone Marrow Cell
· Reprogram	Sendai virus (CytoTune-iPS Reprogramming kit, Invitrogen)
	OCT3/4, SOX2, KLF4, c-MYC

Reference

Rim YA et al. Recent progress of national banking project on homozygous HLA-typed induced pluripotent stem cells in South Korea. J Tissue Eng Regen Med. 2018 Mar;12(3):e1531-e1536.

Mycoplasma contamination test

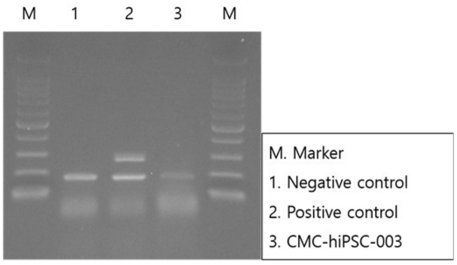

Cell attachment & Morphology

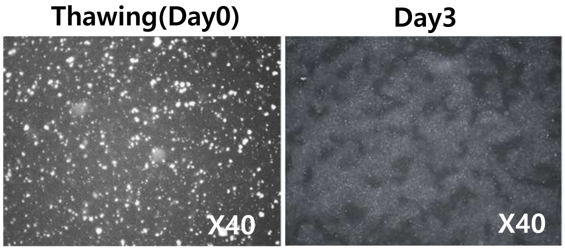

Microbial contamination test

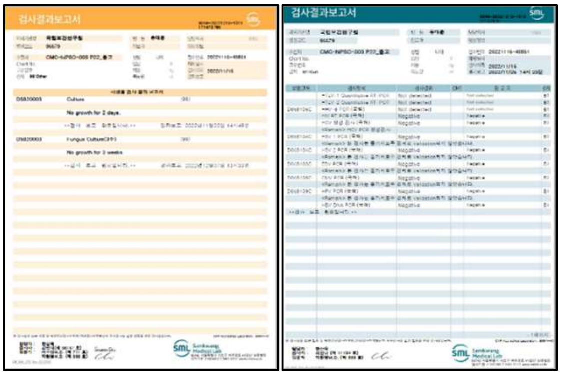

CNV analysis

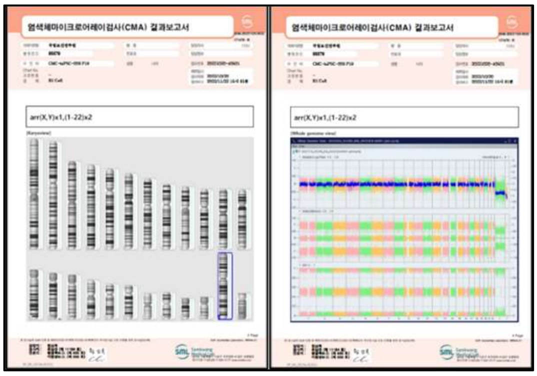

Karyotype(46,XY)

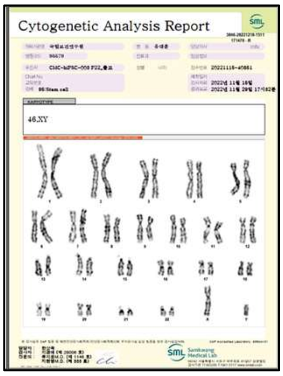

AP staining

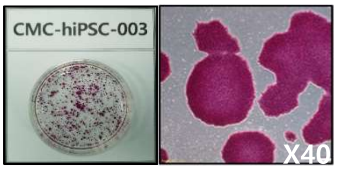

Differentiation

<EB formation>

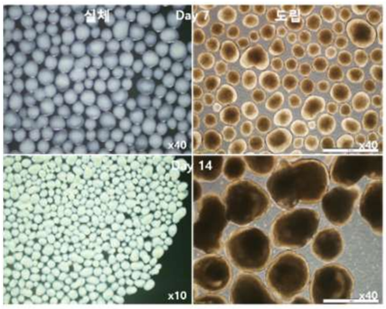

Teratoma

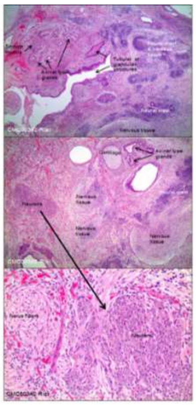

Stem cell marker

<ICC>

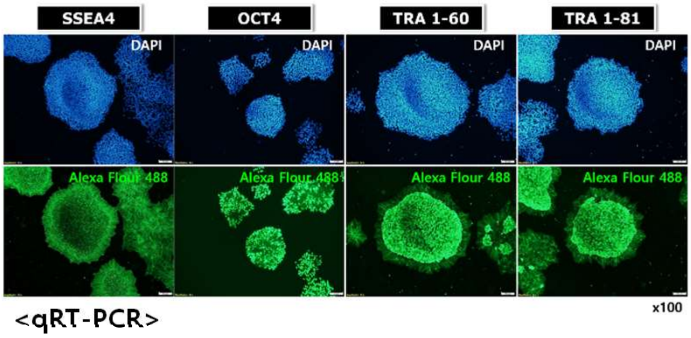

<qRT-PCR>

Gene	Ct Mean
NANOG	20.602
POU5F1(OCT4)	24.884
SOX2	24.125
TERT	25.725
TDGF1	20.218
DNMT3B	20.702
GABRB3	22.074
GDF3	24.149
REX1	24.446
GAPDH	17.975

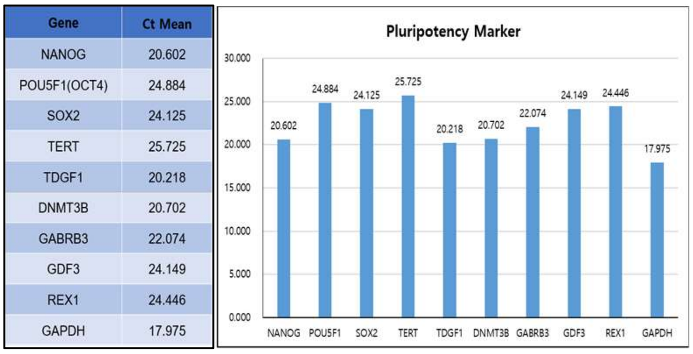

<qRT-PCR>

Gene	Ct Mean
PAX6	24.053
NR2F2	28.156
EMX2OS	27.257
T	27.217
HAND1	27.988
ITGA8	26.372
HNF3b	24.916
AFP	34.867
IHH	27.165
ACTB	17.381
GAPDH	17.291

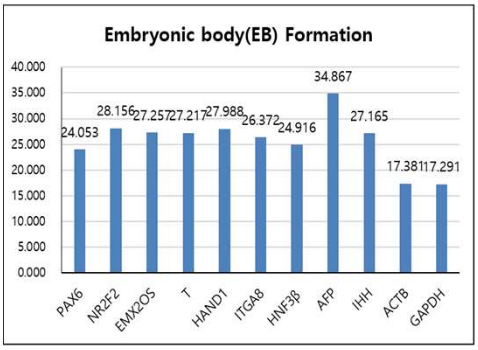

Supplement: Supplementary file 3 — Supplementary Material 3 [file 12964_2025_2172_MOESM3_ESM.pdf]
